# Supplementary material for: From Gondwana to the Yellow Sea, evolutionary diversifications of true toads Bufo sp. in the Eastern Palearctic and a revisit of species boundaries for Asian lineages
Source: eLife. 2022 Jan 28;11:e70494. doi: 10.7554/eLife.70494 (PMC8920510; doi:10.7554/eLife.70494)
Supplement: Supplementary file 1. [file elife-70494-supp1.docx]

**Supplementary file 1A.**

**Type localities of the species members of East Asian *Bufo* discussed in this study.** The information retrieves from electronic catalogues of Amphibian species of the world (Frost, 2021). See the reference list at the end of the supplementary file for the resources mentioned in this table.

| **Names of taxa (taxonomy ID)** | **Type locality** | **Types** | **References** |
| --- | --- | --- | --- |
| *Bufo vulgaris*  Laurenti, 1768 | "Presumably in Europe | By indication the frog(s) illustrated by Rosenhof et al. (1758) | Laurenti (1768) |
| *Bufo gargarizans* Cantor, 1842 | Chusan Island, East China Sea, of northeastern coast of Zhejiang, China | Presumably to be originated in BMNH | Cantor (1842) |
| *Bufo gargarizans* *popei* Matsui, 1986 | San Chiang, Ch'ungan Hsien, Fukien (Fujian), Central China | AMNH 30510, an adult male, altitude 360-450 m (27° 42' N,  117° 47' E), during April and Sept. 1926 by Clifford Pope. | Matsui (1986) |
| *Bufo vulgaris* var. *asiaticus* Steindachner, 1867 | Shanghai, China | (Presumably NHMW) but not mentioned in recent type lists | Steindachner (1867) |
| *Bufo vulgaris* var. *sachalinensis* Nikolsky, 1905 | Sachalin (Sakhalin Island), Russia | Syntypes ZISP 1934–1936 and MNKNU 26290 (perhaps, vicinities of Aleksandrovsk-Sakhalinsky Town, where lived the collector), during 1890 by P. I. Surupnenko. | Nikolsky (1905),  Milto and Barabanov (2011) |
| *Bufo andrewsi* Schmidt, 1925 | Likiang, 8500 feet altitude, Yunnan, China | AMNH 5769 | Schmidt (1925) |
| *Bufo tibetanus* Zarevskij, 1926 | Dza Chyu River, Tibet, plateau of Kham, Qinghai, China | Lectotype: ZISP 2638.2, an adult male (Tibet, Kham, Dsa-tshu River, near the confluence of the river Ba-tshu, about 33.01°N 97.09°E, 3700 m a.s.l.), during August 23-31, 1900 by A. N. Kaznakov. | Zarevskij (1926),  Borkin and Matsui (1986) |
| *Bufo bankorensis* Barbour, 1908 | Bankoro, Central Formosa, Taiwan | MCZ 2432 (2 specimens) | Barbour (1908) |
| *Bufo minshanicus* Stejneger, 1926 | Choni on Tao River, Gansu, China | USNM 68567 | Stejneger (1926) |
| *Bufo bufo miyakonis* Okada, 1931 | Miyakojima, Riu-Kiu (Ryukyu) Islands, Japan | Zool. Inst., Tokyo, no. 2007 | Okada (1931) |
| *Bufo formosus* Boulenger, 1883 | Yokohama, Japan | MNH (2 specimens) | Boulenger (1883) |
| **Bufo praetextatus* Boie, 1826 | Nangasacki, Japan | Presumably to be originated in ZMA | Boie (1826) |
| *Bufo vulgaris japonicus* Temminck and Schlegel, 1838 | Japan | Syntypes include RMNH 2109, 2115-17, 2119, with RMNH 2119A | Temminck & Schlegel (1838) |
| *Bufo torrenticola* Matsui, 1976 | Shiokara-dani Valley, 1400 metres on Mt. Hidegatake, Ohdaigahara, Nara Prefecture, [Honshu I.], Japan | 完模式標本: Am 4202; Ohdaigahara, Nara Prefecture, Japan | Matsui (1976) |
| *Bufo tuberculatus* Zarevskij, 1926 | Mountains Ji-us-shan, to east of lake Kuku-nor, Qinghai, Qinghai, China | Lectotype: ZISP 2634.2, an adult male (Mountains Ji-us-shan, to east of lake Kuku-nor, Qinghai, China, more 3040 m a.s.l.), during August 22-23, 1901 by P. K. Kozlov and A. N. Kaznakov. | Zarevskij (1926), Borkin and Matsui (1986) |
| *Bufo cryptotympanicus* Liu and Hu, 1962 | San-men of Hua-ping, Lung-shen-hsien, altitude 870 m, Kwangsi [= Guangxi]", China | CIB 603507 | Liu & Hu (1962) |
| *Torrentophryne aspinia* Rao and Yang, 1994 | Qingko in Taiping of Yangbi, Yunnan (2100 m) | Not given; KIZ 91005 considered the holotype by Yang, Liu, and Rao, 1996, Zool. Res., Kunming, 17: 354 | Rao & Yang (1994) |
| *Torrentophryne tuberospinia* Yang, Liu, and Rao, 1996 | Dahaoping of Tengchong, Yunnan (1900 m) | KIZ 820645 | Yang et al. (1996) |
| *Bufo pageoti* Bourret, 1937 | Fan-Si-Pan (2500 m)", Lao Cai Province, Vietnam. | MNHNP 1948.125 (formerly LZUH B-145) | Bourret (1937) |
| *Bufo stejnegeri* Schmidt, 1931 | Songdo [= Kaesong], Korea (Chosen) | Holotype: FMNH 11417 | Schmidt (1931) |
| *considered as senior synonym to *Bufo vulgaris japonicus* (Stejneger 1907, Okada 1966). | | | |

**Supplementary file 1B.**

**Cross-references for the oldest age of fossil species used to calibrate divergence times under the fossilised birth-death model.** The collection of the fossil abundance and distribution retrieved from the database of vertebrates (FosFARbase).

| **Species group** | **Fossil minimum age range (Mya)** | **References** |
| --- | --- | --- |
| *Rhinella marina* | 11.80 | Estes and Wassersug (1963) |
| *“Bufo” praevius* (TRMCA of  *Anaxyrus* and *Incilius*) | 20 .00 | Tihen (1951; 1962) |
| *Bufotes* cf. *viridis* | 18.00 | Bailon and Hossini (1990) |
| *Epidalea calamita* | 7.00 | Sanchiz (1997, 1998) |
| *Strauchbufo raddei* | 7.00 | Syromyatnikova (2015) |
| *Bufo bufo* | 9.00 | Rage and Roček (2003) |
| *Bufo gargarizans* | <1.00 | Ratnikov (2001) |

**Supplementary file 1C.**

**Rank of support for topology models to resolve speciation patterns of the Eastern Palearctic *Bufo* species.** We conducted nested sampling analyses on species tree models to evaluate the most likely species tree topology. The Miocene geological events (Model C) was the highest supported factor in driving the speciation of Asian *Bufo* based on the lowest MLE and positive value of Bayes factor obtained.

| **Model** | **Species tree topology scenarios** | **Nested sampling** | | | | **Bayes Factor** | **Rank** |
| --- | --- | --- | --- | --- | --- | --- | --- |
|  |  | **MLE^a^** | **Sqrt (H/N)^b^** | **SD^c^** | **Max ESS** |  |  |
| A | Likelihood based on distributional range and isolation by distance rule | -15310.368 | 8.365 | 8.287 | 1210.783 | -73.416 | 4 |
| B | Likelihood based on life history and adaptive morphological traits | -15360.660 | 8.327 | 8.445 | 1425.379 | -50.292 | 5 |
| C | Likelihood based on Miocene geological events | -15187.432 | 8.186 | 8.552 | 1178.960 | 8.642 | 1 |
| D | Alternative 1 - two independent origins of Eastern Palearctic *Bufo*: East Asian mainland vs. Japanese lineages | -15236.952 | 7.923 | 7.931 | 953.015 | -40.878 | 3 |
| E | Alternative 2 - A single origin of Asian *Bufo* with three monophyletic groups | -15196.074 | 8.301 | 8.690 | 1378.164 | - | 2 |

**Supplementary file 1D.**

**Population genetic analyses and test of population expansion derived on 13 subpopulations of *Bufo* distributed in East Asia.** The statistical analyses inferred from 894 bp of concatenated *CR*-*ND2* of 214 individuals, consisted of gene diversity, mean of pairwise diversity (π), nucleotide diversity, Test of goodness of fit (Harpending’s raggedness), Neutrality Test; Tajima’s D, Li and Fu statistic, and mismatch distribution. Haplotype data had 214 segregating sites, among which 178 were informative.

| **Clade** | **Number of haplotype (n)** | **Species** | **Lineage region** | **Gene diversity ±SD** | **Mean of pairwise diversity (π) ±SD** | **Nucleotide diversity (average over loci)** | **Harpending's raggedness (r) index**  **(*p*–value)** | **Tajima's D (*p*- value)** | **Fu's Fs test**  **(*p*-value)** | **Pattern of mismatch distribution** |
| --- | --- | --- | --- | --- | --- | --- | --- | --- | --- | --- |
| 3 | 2 | *Bufo stejnegeri* | Korean Peninsula | 0.67 ±0.31 | 4.67 ±3.13 | 0.01 ±0.01 | 1.00  (*p*=0.31) | 0.00 (*p*=1.00) | 2.88 (*p*=0.86) | unimodal |
| 1 | 3 | *Bufo japonicus formosus* | Japan | 1.00 ±0.10 | 20.00 ±12.31 | 0.02 ±0.02 | 1.11  (*p*=1.00) | 0.00 (*p*=1.00) | 1.86 (*p*=0.54) | multimodal |
| 2 | 3 | *Bufo japonicus japonicus* | Japan | 1.00 ± 0.27 | 8.00 ±5.13 | 0.01 ±0.01 | 1.11  (*p*=0.05) | 0.00 (*p*=1.00) | 0.9 (*p*=0.44) | multimodal |
| 4 | 2 | *Bufo tibetanus* | western Mainland | 1.00 ± 0.50 | 8.00 ±6.00 | 0.01 ±0.01 | Unidentified | 0.00 (*p*=1.00) | 2.08 (*p*=0.54) | unimodal |
| 4 | 12 | *Bufo andrewsi* | western Mainland | 0.99 ±0.04 | 32.15 ±15.02 | 0.04 ±0.02 | 0.03  (*p*=0.51) | 1.10 (*p*=0.92) | *–*0.43 (*p*=0.33) | multimodal |
| 5 | 7 | *Bufo gargarizans* | western Mainland | 0.82 ±0.12 | 20.27 ±9.72 | 0.02 ±0.01 | 0.07  (*p*=0.28) | *–*1.22 (*p*=0.13) | 8.96 (*p*=0.91) | multimodal |
| 5 | 5 | *Bufo gargarizans* | central Mainland | 1.00 ±0.13 | 44.40 ±23.35 | 0.05 ±0.03 | 0.14  (*p*=0.93) | 0.62 (*p*=0.72) | 1.41 (*p*=0.49) | multimodal |
| 5 | 25 | *Bufo gargarizans* | southeastern Mainland | 0.98 ±0.01 | 13.41 ±6.19 | 0.02 ±0.01 | 0.01  (*p*=0.68) | *–*1.05 (*p*=0.15) | *–*6.15  (*p*=0.03) | multimodal |
| 4 | 2 | *Bufo andrewsi* | southeastern Mainland | 0.67 ±0.20 | 37.33 ±20.76 | 0.04 ±0.03 | 1.00  (*p*=0.07) | 2.31 (*p*=1.0) | 9.22 (*p*=1.0) | Unidentified |
| 5 | 3 | *Bufo bankorensis* | Taiwan Island | 0.83 ±0.22 | 23.67 ±13.29 | 0.03 ±0.02 | 0.42  (*p*=0.47) | *–*0.80 (*p*=0.17) | 4.13 (*p*=0.90) | multimodal |
| 5 | 12 | *Bufo gargarizans* | north eastern Mainland | 0.94 ±0.05 | 17.43 ±8.21 | 0.02 ±0.01 | 0.04  (*p*=0.46) | *–*1.20 (*p*=0.15) | *–*0.38 (*p*=0.40) | multimodal |
| 6 | 26 | *Bufo sachalinensis* cf. *sachalinensis* | Korean Peninsula | 0.88 ±0.02 | 4.76 ± 2.34 | 0.01 ±0.003 | 0.05 (*p*=0.0) | *–*0.67 (*p*=0.29) | *–*6.33 (*p*=0.05) | multimodal |
| 6 | 7 | *Bufo sachalinensis sachalinensis* | Amur River Basin | 0.50 ±0.12 | 9.75 ± 4.63 | 0.01 ±0.006 | 0.24 (*p*=1.0) | *–*1.53 (*p*=0.05) | 5.77 (*p*=0.98) | multimodal |

**Supplementary file 1E.**

**AMOVA statistic on six clades obtained through mitochondrial phylogenetic tree**. The results (%) show a differentiation for different populations, different individual and within individual.

| **Source** | **Degree of freedom (df)** | **Sum of Square** | **Estimated variance** | **Variance (%)** |
| --- | --- | --- | --- | --- |
| Among populations | 5 | 2218.164 | 14.039 | 55.48 |
| Among Individuals | 7 | 564.393 | 5.588 | 22.08 |
| Within Individuals | 213 | 1209.726 | 5.679 | 22.44 |
| Total | 225 | 3992.283 | 25.307 |  |

**Supplementary file 1F.**

CLUMPP analysis for best cluster (K = 2) for STRUCTURE analysis.

| **Population** | | **Admixture** | |
| --- | --- | --- | --- |
|  |  | **Portion 1** | **Portion 2** |
| western Mainland | | 0.999 | 0.001 |
| central Mainland | | 0.830 | 0.170 |
| southeastern Mainland | | 0.952 | 0.048 |
| northeastern Mainland | | 0.955 | 0.005 |
| Korean Peninsula | | 0.033 | 0.967 |
| Amur River Basin | | 0.017 | 0.983 |
| Taiwan Island | | 0.005 | 0.995 |
| Japanese Archipelago | | 0.975 | 0.025 |
| **GPopulation** | **Individual** | **Cluster (K = 2)** | |
|  |  | **PPortion 1** | **PPortion 2** |
| western Mainland | BR121_WC | 0.006 | 0.994 |
| western Mainland | BR672_WC | 0.004 | 0.996 |
| central Mainland | BR651_CC | 0.032 | 0.968 |
| central Mainland | BR652_CC | 0.997 | 0.003 |
| central Mainland | BR653_CC | 0.004 | 0.996 |
| central Mainland | BR667_CC | 0.008 | 0.992 |
| central Mainland | BR710_CC | 0.004 | 0.996 |
| central Mainland | BR712_CC | 0.004 | 0.996 |
| southeastern Mainland | 18BGC21SY_SEC | 0.008 | 0.992 |
| southeastern Mainland | 18BGC31H_SEC | 0.001 | 0.999 |
| southeastern Mainland | 18BGC32H_SEC | 0.002 | 0.998 |
| southeastern Mainland | 18BGC34H_SEC | 0.039 | 0.961 |
| southeastern Mainland | 18BGC35H_SEC | 0.005 | 0.995 |
| southeastern Mainland | 18BGC36H_SEC | 0.01 | 0.99 |
| southeastern Mainland | 18BgC400_SEC | 0.018 | 0.982 |
| southeastern Mainland | 18BgC401J_SEC | 0.008 | 0.992 |
| southeastern Mainland | CLPT317_SEC | 0.003 | 0.997 |
| southeastern Mainland | CLPT325_SEC | 0.006 | 0.994 |
| southeastern Mainland | CLPT331_SEC | 0.87 | 0.13 |
| southeastern Mainland | CLPT351_SEC | 0.004 | 0.996 |
| southeastern Mainland | CLPT364_SEC | 0.008 | 0.992 |
| southeastern Mainland | CLPT398_SEC | 0.003 | 0.997 |
| southeastern Mainland | CLPT403_SEC | 0.001 | 0.999 |
| southeastern Mainland | CLPT407_SEC | 0.005 | 0.995 |
| southeastern Mainland | SHS7B13_SEC | 0.008 | 0.992 |
| northeastern Mainland | BR505_NEC | 0.001 | 0.999 |
| northeastern Mainland | BR517_NEC | 0.011 | 0.989 |
| northeastern Mainland | BR518_NEC | 0.003 | 0.997 |
| northeastern Mainland | BR568_NEC | 0.004 | 0.996 |
| northeastern Mainland | 17BgC002_NEC | 0.014 | 0.986 |
| Korean Peninsula | 19Stj002 | 0.996 | 0.004 |
| Korean Peninsula | 19Stj003 | 0.989 | 0.011 |
| Korean Peninsula | BGD41 | 0.898 | 0.102 |
| Korean Peninsula | BGD45 | 0.992 | 0.008 |
| Korean Peninsula | BGD515 | 0.998 | 0.002 |
| Korean Peninsula | BGD524 | 0.992 | 0.008 |
| Korean Peninsula | BGE31 | 0.973 | 0.027 |
| Amur River Basin | BR794_Ru | 0.993 | 0.007 |
| Amur River Basin | BR12_RuSk | 0.986 | 0.014 |
| Taiwan Island | 19Bb0017 | 0.998 | 0.002 |
| Taiwan Island | 19Bb0178 | 0.997 | 0.003 |
| Taiwan Island | 19Bb0261 | 0.984 | 0.016 |
| Japan Archipelago | 18BjJ015 | 0.034 | 0.966 |
| Japan Archipelago | 18BjJ017_f | 0.005 | 0.995 |

**Supplementary file 1G.**

**Dating of the origin of the *Bufo* genus in East Asia.** Here we determined the divergence time for all relevant nodes related to ancestral dispersion routes and the most probable ancestral regions.

| **Divergence time**  **(Mean**  **Age/ Mya)** | **Relevant nodes** | **Most probable ancestral areas** | **Probability of ancestral areas (%)** | **Events matrix** | |
| --- | --- | --- | --- | --- | --- |
|  |  |  |  | **Dispersal factor** | **Vicariance factor** |
| 20.38 | Root node of tree (Earliest split off between East Asian *Bufo* from the genus *Bufotes*) | West Palearctic to central East Asian mainland | 55.86 | 1.00 | 1.00 |
| 10.48 | Emergence of East Asian *Bufo* | Southwestern, central, northeastern Mainland to Korean Peninsula | 46.32 | 1.00 | 1.00 |
| 8.36 | Stem clade of East Asian mainland lineage of *Bufo* | Central, northeastern Mainland to Korean Peninsula | 55.91 | 2.00 | 0.00 |
| 7.71 | Stem clade of Japanese *Bufo* | Japan | 62.48 | 0.00 | 0.00 |
| 6.82 | Emergence of East Asian mainland lineage of *B. gargarizans* | Central to northeastern Mainland to Korean Peninsula | 86.64 | 1.00 | 1.00 |
| 5.20 | Stem clade of *B. tibetanus* and *B. andrewsi* | Western to central mainland of East Asia | 69.31 | 1.00 | 0.00 |
| 3.31 | Crown clade of Korean *B. stejnegeri* | Korean Peninsula | 100.00 | 0.00 | 0.00 |
| 3.11 | Emergence of southeastern Mainland clade of *B. g. gargarizans* | Central to northeastern Mainland of East Asia | 89.22 | 1.00 | 0.00 |
| 2.85 | Stem clade of southwestern Mainland lineage: *B.* *minshanicus* and *B. gargarizans* inhabited high latitudes | Western to central mainland of East Asia | 51.57 | 2.00 | 0.00 |
| 1.30 | Crown node of endemic Taiwan Island *B. bankorensis* | central, southeastern Mainland to Taiwan Island | 99.87 | 0.00 | 1.00 |
| 1.91 | Septentrional East Asia lineages of *Bufo* | Korean Peninsula- Amur River Basin | 93.96 | 0.00 | 1.00 |
| 1.53 | Crown clade of Korean *B. sachalinensis* cf. *sachalinensis* | Korean Peninsula | 84.47 | 0.00 | 0.00 |
| 1.02 | Crown clade of Amur River Basin *B. sachalinensis sachalinensis* | Amur River Basin | 87.94 | 0.00 | 0.00 |

**Supplementary file 1H.**

**Migration patterns among the septentrional populations of East Asian mainland *Bufo*.** We inferred the migration patterns for three populations of East Asian *Bufo* (northeastern Mainland, Amur River Basin and Korean Peninsula) and two populations of *B. stejnegeri* on the Korean Peninsula. Maximum likelihood estimates the average of effective population size (Θ) and 25-97.5% confidence intervals (in parentheses), with the computed gene flow rates between the regions characterised on the mtDNA data (2*Nm*) and unlinked nuDNA multi-loci (4*Nm*).

| **Species** | **Population (i)** | **2Nm** | | | | | | **4Nm** | | | | **Mean migration rate (Θ/CI)** |
| --- | --- | --- | --- | --- | --- | --- | --- | --- | --- | --- | --- | --- |
|  |  | Θ (CI) | NM 🡪 i | ARB 🡪 i | | KP 🡪 i | | Θ | NM 🡪 i | ARB🡪 i | KP 🡪 i |  |
| *Bufo gargarizans gargarizans* | Northeastern Mainland (NM) | 0.0025  (0.0006 ‒ 0.0064) | N/A | 0.425 | | 0.316 | | 0.0002 (0.0000-0.0004) | N/A | 0.099 | 0.112 | 0.00135  (0.0003 ‒ 0.0034) |
|  |  |  |  |  |  |  |  |  |  |  |  |  |
| *Bufo sachalinensis sachalinensis* | Amur River Basin (ARB) | 0.0012  (0.0006 ‒ 0.0025) | 0.251 | N/A | | 0.268 | | 0.0196 (0.0000-0.0106) | 11.963 | N/A | 12.499 | 0.0104  (0.0003 ‒ 0.00655) |
| *Bufo sachalinensis* cf. *sachalinensis* | Korean Peninsula (KP) | 0.0018  (0.0009 ‒ 0.0029) | 0.174 | 0.272 | | N/A | | 0.00006 (0.0000-0.00017) | 0.038 | 0.304 | N/A | 0.00093  (0.00045 ‒ 0.00154) |
| **Species** | **Population (i)** | **Θ (CI)** | **Northern 🡪 i** | | **Southern 🡪 i** | |  |  |  |  |  |  |
| *Bufo stejnegeri* | North | 0.00002  (0.000 ‒ 0.0001) | N/A | 0.005 | |  | |  |  |  |  |  |
|  | South | 0.00008  (0.0000 ‒ 0.0002) | 0.026 | N/A | |  | |  |  |  |  |  |

**Supplementary file 1I.**

**Species delimitation models and their respective scenarios.** We tested each model using the coalescent approach and ranked them based on the probability of a single independent evolutionary lineage determined by the highest to lowest value of the Marginal likelihood estimation (MLE) and positive to negative values of the Bayes Factor.

| **Models** | **Species delimitation scenario** | **Marginal likelihood Estimation L (MLE)** | **Ln MLE** | **Bayes Factor (BF)**  **BF = 2 x (MLE _model 1_ – MLE _model 2_)** | **Rank of support to most probable model** |
| --- | --- | --- | --- | --- | --- |
| A | 7 species - splitting all East Asian mainland members of *B. gargarizans* complex with single evolutionary lineages, but lumping *B. sachalinensis* cf. *sachalinensis* and *B. s. sachalinensis* as a single species | – 502.801 | 6.220 | 3.198 | 1 |
| B | 8 species assigned - Splitting all members of *B. gargarizans* complex and *B. j. japonicus* subspecies into a single species | – 504.400 | 6.223 | - | 2 |
| C | 5 species assigned - Lumping *B. andrewsi* with *gargarizans*, lumping *B. sachalinensis* cf. *sachalinensis* with *B. s. sachalinensis*, merging the subspecies of *B. j. japonicus* into a single lineage. | – 506.013 | 6.227 | – 3.226 | 3 |
| D | 5 species assigned - Splitting the continental species: *B. bankorensis, B. j. formosus,* and *B. j. japonicus* as independent species, lumping the mainland members into a single species: *B. andrewsi*, *B. gargarizans*, *B. sachalinensis* cf. *sachalinensis* and *B. s. sachalinensis*. | – 531.009 | 6.275 | – 24.996 | 4 |
| E | 4 species assigned - Lumping mainland *Bufo*: *B. andrewsi*, *B. bankorensis* into a single species of *B. gargarizans*, splitting the mainland from septentrional Eastern Asia *Bufo:* *B. sachalinensis cf. sachalinensis* and *B. s. sachalinensis* | – 532.218 | 6.277 | – 1.209 | 5 |
| G | 6 species assigned - lumping *B. bankorensis* into *B. gargarizans* unit and splitting *B. andrewsi* as a single species. Lumping *B. sachalinensis* cf. *sachalinensis* into *B. s. sachalinensis* unit and splitting the subspecies of *B. japonicus* | – 533.603 | 6.280 | – 1.385 | 6 |
|  |  |  |  |  |  |
| F | 3 species assigned - Lumping of all *B. gargarizans* complex members into a single species, merging of two subspecies of *B. japonicus* into a unit | – 537.695 | 6.287 | – 4.092 | 7 |
| H | 6 species assigned - Lumping *B. sachalinensis* with east Asian *B. gargarizans*, while splitting *B. andrewsi,* *B. bankorensis,* and *B. sachalinensis* as independent species | – 1209.453 | 7.098 | – 671.758 | 8 |

**Supplementary file 1J.**

Sampling locations of East Asian *Bufo* spp. and the Genbank accession numbers for all sequences used in the species tree and phylogenetic analyses. See the reference list at the end of the supplementary file for the resources mentioned in this table.

| **Species** | **Voucher/ specimen ID** | **Lat** | **Lon** | **Localities** | ***16S*** | ***CR*  (D-loop)** | ***ND2*** | ***POMC*** | ***RAG-1*** | ***Rho*** | ***CXCR4*** | **Reference** |
| --- | --- | --- | --- | --- | --- | --- | --- | --- | --- | --- | --- | --- |
| *Bufo bankorensis* | 19BbTw001 | 23.913 | 120.879 | Taiwan island: Lienhuachih Reserve Forest, Taichung |  | MW081664 | MW467646 |  |  |  |  | This study |
| *Bufo bankorensis* | 19BbTw002 | 23.913 | 120.879 | Taiwan island: Lienhuachih Reserve Forest, Taichung |  | MW081665 | MW467647 |  |  |  |  | This study |
| *Bufo bankorensis* | 19BbTw003 | 23.913 | 120.879 | Taiwan island: Lienhuachih Reserve Forest, Taichung |  | MW081666 |  |  |  |  |  | This study |
| *Bufo bankorensis* | BB0005 | 25.19 | 121.56 | Taiwan island: Yangminshan National Park |  | MW081668 | MW467651 | MW489915 |  |  |  | This study |
| *Bufo bankorensis* | BB0017 | 25.19 | 121.56 | Taiwan island: Yangminshan National Park |  | MW081669 |  | MW489916 | MW489986 | MW507752 |  | This study |
| *Bufo bankorensis* | BB0178 | 25.19 | 121.56 | Taiwan island: Yangminshan National Park |  | MW081670 |  | MW489917 | MW489987 | MW507753 |  | This study |
| *Bufo bankorensis* | BB0261 | 25.19 | 121.56 | Taiwan island: Yangminshan National Park |  | MW081671 | MW467652 | MW489918 | MW489988 | MW507754 |  | This study |
| *Bufo stejnegeri* | 19Stj001 | 35.3 | 127.61 | Republic of Korea: Jirisan |  | MW507738 | MW467648 | MW489912 | MW489983 |  |  | This study |
| *Bufo stejnegeri* | 19Stj002 | 35.3 | 127.61 | Republic of Korea: Jirisan |  | MW507736 | MW467649 | MW489913 | MW489984 | MW507750 |  | This study |
| *Bufo stejnegeri* | 19Stj003 | 35.3 | 127.61 | Republic of Korea: Jirisan |  | MW507737 | MW467650 | MW489914 | MW489985 | MW507751 |  | This study |
| *Bufo stejnegeri* | 15WHB3 | 37.65 | 128.32 | Republic of Korea: Pyeongchang |  | MW081603 |  |  |  |  |  | This study |
| *Bufo stejnegeri* | 15WHB5 | 37.65 | 128.32 | Republic of Korea: Pyeongchang |  | MW081604 |  |  |  |  |  | This study |
| *Bufo stejnegeri* | 15WHB7 | 37.65 | 128.32 | Republic of Korea: Pyeongchang |  | MW081605 |  |  |  |  |  | This study |
| *Bufo stejnegeri* | 15WHB8 | 37.65 | 128.32 | Republic of Korea: Pyeongchang |  | MW081606 |  |  |  |  |  | This study |
| *Bufo stejnegeri* | 15WHB9 | 37.65 | 128.32 | Republic of Korea: Pyeongchang |  | MW081607 |  |  |  |  |  | This study |
| *Bufo stejnegeri* | 15WHB10 | 37.65 | 128.32 | Republic of Korea: Pyeongchang |  | MW081608 |  |  |  |  |  | This study |
| *Bufo stejnegeri* | 15WHB12 | 37.65 | 128.32 | Republic of Korea: Pyeongchang |  | MW081609 |  |  |  |  |  | This study |
| *Bufo stejnegeri* | 15WHB13 | 37.65 | 128.32 | Republic of Korea: Pyeongchang |  | MW081610 |  |  |  |  |  | This study |
| *Bufo stejnegeri* | 15WHB14 | 37.65 | 128.32 | Republic of Korea: Pyeongchang |  | MW081611 |  |  |  |  |  | This study |
| *Bufo japonicus formosus* | 18BjJ001 | 35.69 | 140.03 | Japan: Chiba |  | MW081649 |  |  |  |  |  | This study |
| *Bufo japonicus formosus* | 18BjJ002 | 35.69 | 140.03 | Japan: Chiba |  | MW081650 |  |  |  |  |  | This study |
| *Bufo japonicus formosus* | 18BjJ003 | 35.69 | 140.03 | Japan: Chiba |  | MW081651 |  | MW489906 |  |  |  | This study |
| *Bufo japonicus formosus* | 18BjJ005 | 35.69 | 140.03 | Japan: Chiba |  | MW081652 |  | MW489907 |  |  |  | This study |
| *Bufo japonicus formosus* | 18BjJ006 | 37.65 | 140.03 | Japan: Chiba |  | MW081653 |  |  |  |  |  | This study |
| *Bufo japonicus formosus* | 18BjJ007 | 37.65 | 140.03 | Japan: Chiba |  | MW081654 |  |  |  |  |  | This study |
| *Bufo japonicus formosus* | 18BjJ008 | 37.65 | 140.03 | Japan: Chiba |  | MW081655 |  |  |  |  |  | This study |
| *Bufo japonicus japonicus* | 18BjJ009 | 35.42 | 139.42 | Japan: Kansen-en Park,Tokyo |  | MW081656 | MW467640 |  |  |  |  | This study |
| *Bufo japonicus japonicus* | 18BjJ010 | 35.42 | 139.42 | Japan: Kansen-en Park,Tokyo |  | MW081657 |  |  |  |  |  | This study |
| *Bufo japonicus japonicus* | 18BjJ011 | 35.42 | 139.42 | Japan: Kansen-en Park,Tokyo |  | MW081658 |  | MW489908 |  |  |  | This study |
| *Bufo japonicus japonicus* | 18BjJ012 | 35.42 | 139.42 | Japan: Kansen-en Park,Tokyo |  |  |  |  |  |  |  | This study |
| *Bufo japonicus japonicus* | 18BjJ013 | 35.42 | 139.42 | Japan: Kansen-en Park,Tokyo |  | MW081659 | MW467641 | MW489909 |  |  |  | This study |
| *Bufo japonicus japonicus* | 18BjJ015 | 35.39 | 139.41 | Japan: Saigo-yama ParkTokyo |  | MW081660 | MW467642 | MW489910 | MW489980 | MW507748 |  | This study |
| *Bufo japonicus japonicus* | 18BjJ016 | 35.39 | 139.41 | Japan: Saigo-yama ParkTokyo |  | MW081661 | MW467643 |  | MW489981 |  |  | This study |
| *Bufo japonicus japonicus* | 18BjJ017 | 36.32 | 139.2 | Japan: Tokyo Uni of Agriculture and Technology Research forest, Tokyo |  | MW081662 | MW467644 | MW489910 | MW489982 | MW507749 |  | This study |
| *Bufo japonicus japonicus* | 18BjJ018 | 36.32 | 139.2 | Japan: Tokyo Uni of Agriculture and Technology Research forest, Tokyo |  | MW081663 | MW467645 |  |  |  |  | This study |
| *Bufo japonicus formosus* | 19JHBJ001 | 41.76 | 140.7 | Japan: Mt. Hakotate, Hokkaido |  | MW081667 |  |  |  |  |  | This study |
| *Bufo gargarizans* | 17BgC001 | 41.84 | 123.59 | China: Shenyang |  |  | MW467604 |  |  |  |  | This study |
| *Bufo gargarizans* | 17BgC002 | 41.84 | 123.59 | China: Shenyang |  | MW081612 |  | MW489888 | MW489967 |  |  | This study |
| *Bufo gargarizans* | 17BgC003 | 41.84 | 123.59 | China: Shenyang |  | MW081613 | MW467605 | MW489889 |  |  |  | This study |
| *Bufo gargarizans* | 17BgC004 | 41.84 | 123.59 | China: Shenyang |  | MW081614 | MW467606 |  |  |  |  | This study |
| *Bufo gargarizans* | 17BgC005 | 31.85 | 118.91 | China: Nanjing Airport |  | MW081615 | MW467607 | MW489890 |  |  |  | This study |
| *Bufo gargarizans* | 17BgC006 | 31.85 | 118.91 | China: Nanjing Airport |  | MW081616 |  |  |  |  |  | This study |
| *Bufo gargarizans* | 17BgC007 | 31.85 | 118.91 | China: Nanjing Airport |  | MW081617 |  |  |  |  |  | This study |
| *Bufo gargarizans* | 17BgC008 | 31.85 | 118.91 | China: Nanjing Airport |  | MW081618 | MW467608 | MW489891 |  |  |  | This study |
| *Bufo gargarizans* | 18Bg404 | 36.75 | 116.98 | China: Northern Jinan |  | MW081643 |  |  |  |  |  | This study |
| *Bufo s. sachalinensis* | 18Bgar001 | 43.22 | 132.92 | Russia: Tigrovi |  | MW081647 |  |  |  |  |  | This study |
| *Bufo s. sachalinensis* | 18Bgar002 | 43.22 | 132.92 | Russia: Tigrovi |  | MW081648 |  |  |  |  |  | This study |
| *Bufo gargarizans* | 18BgC003J | 31.91 | 118.81 | China: Jiangsu |  | MW081633 | MW467609 |  |  |  |  | This study |
| *Bufo gargarizans* | 18BgC020 | 31.6 | 118.7 | China: Ma'anshan |  |  | MW467610 |  |  |  |  | This study |
| *Bufo gargarizans* | 18BgC021SY | 31.86 | 117.28 | China: Southern Yangtze, Hefei |  | MW081620 |  | MW489892 | MW489968 | MW507740 |  | This study |
| *Bufo gargarizans* | 18BGC025SY | 31.86 | 117.28 | China: Southern Yangtze, Hefei |  | MW081621 | MW467611 | MW489893 | MW489969 |  |  | This study |
| *Bufo gargarizans* | 18BgC026 | 32.18 | 117.14 | China: Hefei |  | MW081622 | MW467612 |  |  |  |  | This study |
| *Bufo gargarizans* | 18BgC027 | 32.18 | 117.14 | China: Hefei |  | MW081623 | MW467613 |  |  |  |  | This study |
| *Bufo gargarizans* | 18BgC028H | 32.18 | 117.14 | China: Hefei |  | MW081624 | MW467614 |  |  |  |  | This study |
| *Bufo gargarizans* | 18BgC029H | 32.18 | 117.14 | China: Hefei |  | MW081625 | MW467615 | MW489894 | MW489970 |  |  | This study |
| *Bufo gargarizans* | 18BgC030H | 32.18 | 117.14 | China: Hefei |  | MW081626 | MW467616 | MW489895 | MW489971 |  |  | This study |
| *Bufo gargarizans* | 18BgC031H | 32.18 | 117.14 | China: Hefei |  | MW081627 | MW467617 | MW489896 | MW489972 | MW507741 |  | This study |
| *Bufo gargarizans* | 18BgC032H | 32.18 | 117.14 | China: Hefei |  | MW081628 | MW467618 | MW489897 | MW489973 | MW507742 |  | This study |
| *Bufo gargarizans* | 18BgC033H | 32.18 | 117.14 | China: Hefei |  | MW081629 | MW467619 | MW489898 |  |  |  | This study |
| *Bufo gargarizans* | 18BgC034H | 32.18 | 117.14 | China: Hefei |  | MW081630 | MW467620 | MW489899 | MW489974 | MW507743 |  | This study |
| *Bufo gargarizans* | 18BgC035H | 32.18 | 117.14 | China: Hefei |  | MW081631 | MW467621 | MW489900 | MW489975 | MW507744 |  | This study |
| *Bufo gargarizans* | 18BgC036H | 32.18 | 117.14 | China: Hefei |  | MW081632 | MW467622 | MW489901 | MW489976 | MW507745 |  | This study |
| *Bufo gargarizans* | 18BgC037H | 32.18 | 117.14 | China: Hefei |  | MW081633 | MW467623 | MW489902 |  |  |  | This study |
| *Bufo gargarizans* | 18BgC038 | 32.18 | 117.14 | China: Hefei |  | MW081634 | MW467624 |  |  |  |  | This study |
| *Bufo gargarizans* | 18BgC040 | 32.18 | 117.14 | China: North Hefei |  | MW081635 | MW467625 |  |  |  |  | This study |
| *Bufo gargarizans* | 18BgC041 | 32.18 | 117.14 | China: North Hefei |  | MW081636 | MW467626 |  |  |  |  | This study |
| *Bufo gargarizans* | 18BgC042 | 32.18 | 117.14 | China: North Hefei |  | MW081637 | MW467627 |  |  |  |  | This study |
| *Bufo gargarizans* | 18BgC045 | 30.42 | 114.51 | China: Drafo Mountain, Wuhan |  | MW081638 |  |  |  |  |  | This study |
| *Bufo gargarizans* | 18BgC049 | 31.45 | 114.86 | China: Northern Wuhan |  | MW081639 | MW467628 |  |  |  |  | This study |
| *Bufo gargarizans* | 18BgC400 | 36.75 | 116.98 | China: Jinan |  | MW081640 | MW467629 | MW489903 |  | MW507746 |  | This study |
| *Bufo gargarizans* | 18BgC401J | 36.75 | 116.98 | China: Jinan |  | MW081641 | MW467630 | MW489904 | MW489977 | MW507747 |  | This study |
| *Bufo gargarizans* | 18BgC402 | 36.75 | 116.98 | China: Jinan |  | MW081642 | MW467631 | MW489905 | MW489978 |  |  | This study |
| *Bufo gargarizans* | 18BgC404 | 36.75 | 116.98 | China: Jinan |  | MW081643 | MW467632 |  |  |  |  | This study |
| *Bufo gargarizans* | 18BgCN001 | 36.75 | 116.98 | China: Jinan |  | MW081644 | MW467633 |  |  |  |  | This study |
| *Bufo gargarizans* | 18BgN002 | 36.75 | 116.98 | China: Jinan |  |  | MW467634 |  |  |  |  | This study |
| *Bufo gargarizans* | 18BgNL003 | 36.75 | 116.98 | China: Jinan |  | MW081645 |  |  |  |  |  | This study |
| *Bufo gargarizans* | 18BgNL004 | 36.75 | 116.98 | China: Jinan |  |  | MW467635 |  |  |  |  | This study |
| *Bufo s. sachalinensis* | 18BgR001 | 46.57 | 134.68 | Russia: V. pereval, Primosky |  |  | MW467637 |  |  |  |  | This study |
| *Bufo s. sachalinensis* | 18BgR002 | 46.57 | 134.68 | Russia: V. pereval, Primosky |  |  | MW467638 |  |  |  |  | This study |
| *Bufo s. sachalinensis* | 18BgR003 | 46.57 | 134.68 | Russia: V. pereval, Primosky |  |  | MW467639 |  |  |  |  | This study |
| *Bufo s. sachalinensis* | 18BgR010 | 46.57 | 134.68 | Russia: V. pereval, Primosky |  |  |  |  | MW489979 |  |  | This study |
| *Bufo gargarizans* | 18BgPJD001 | 36.75 | 116.98 | China: Dandong |  | MW081646 | MW467636 |  |  |  |  | This study |
| *Bufo gargarizans* | 18BgPJD002 | 36.75 | 116.98 | China: Dandong |  |  |  |  |  |  |  | This study |
| *Bufo sachalinensis* cf. *sachalinensis* | BGA11 | 37.87 | 126.86 | Republic of Korea: Dongducheon |  | MW081672 | MW467653 |  |  |  |  | This study |
| *Bufo sachalinensis* cf. *sachalinensis* | BGA12 | 37.87 | 126.86 | Republic of Korea: Dongducheon |  | MW081673 | MW467654 |  |  |  |  | This study |
| *Bufo sachalinensis* cf. *sachalinensis* | BGA13 | 37.87 | 126.86 | Republic of Korea: Dongducheon |  | MW081674 | MW467655 |  |  |  |  | This study |
| *Bufo sachalinensis* cf. *sachalinensis* | BGA14 | 37.87 | 126.86 | Republic of Korea: Dongducheon |  | MW081675 | MW467656 |  |  |  |  | This study |
| *Bufo sachalinensis* cf. *sachalinensis* | BGA15 | 37.87 | 126.86 | Republic of Korea: Dongducheon |  | MW081676 | MW467657 |  | MW489989 |  |  | This study |
| *Bufo sachalinensis* cf. *sachalinensis* | BGA16 | 37.87 | 126.86 | Republic of Korea: Dongducheon |  | MW081677 | MW467658 |  |  |  |  | This study |
| *Bufo sachalinensis* cf. *sachalinensis* | BGA17 | 37.87 | 126.86 | Republic of Korea: Dongducheon |  |  | MW467659 |  |  |  |  | This study |
| *Bufo sachalinensis* cf. *sachalinensis* | BGB11 | 36.67 | 126.64 | Republic of Korea: Chungcheongnam-do |  | MW081678 | MW467660 |  |  |  |  | This study |
| *Bufo sachalinensis* cf. *sachalinensis* | BGB12 | 36.67 | 126.64 | Republic of Korea: Chungcheongnam-do |  | MW081679 | MW467661 |  |  |  |  | This study |
| *Bufo sachalinensis* cf. *sachalinensis* | BGB13 | 36.67 | 126.64 | Republic of Korea: Chungcheongnam-do |  | MW081680 | MW467662 |  |  |  |  | This study |
| *Bufo sachalinensis* cf. *sachalinensis* | BGB14 | 36.67 | 126.64 | Republic of Korea: Chungcheongnam-do |  | MW081681 | MW467663 |  |  |  |  | This study |
| *Bufo sachalinensis* cf. *sachalinensis* | BGB15 | 36.67 | 126.64 | Republic of Korea: Chungcheongnam-do |  | MW081682 |  |  |  |  |  | This study |
| *Bufo sachalinensis* cf. *sachalinensis* | BGB16 | 36.67 | 126.64 | Republic of Korea: Chungcheongnam-do |  | MW081683 | MW467664 |  |  |  |  | This study |
| *Bufo sachalinensis* cf. *sachalinensis* | BGB17 | 36.67 | 126.64 | Republic of Korea: Chungcheongnam-do |  | MW081684 |  |  |  |  |  | This study |
| *Bufo sachalinensis* cf. *sachalinensis* | BGB18 | 36.67 | 126.64 | Republic of Korea: Chungcheongnam-do |  | MW081685 | MW467665 |  |  |  |  | This study |
| *Bufo sachalinensis* cf. *sachalinensis* | BGB19 | 36.67 | 126.64 | Republic of Korea: Chungcheongnam-do |  | MW081686 | MW467666 |  |  |  |  | This study |
| *Bufo sachalinensis* cf. *sachalinensis* | BGB21 | 36.65 | 127.33 | Republic of Korea: Cheongju |  | MW081687 |  |  |  |  |  | This study |
| *Bufo sachalinensis* cf. *sachalinensis* | BGB22 | 36.65 | 127.33 | Republic of Korea: Cheongju |  | MW081688 | MW467667 |  |  |  |  | This study |
| *Bufo sachalinensis* cf. *sachalinensis* | BGB23 | 36.65 | 127.33 | Republic of Korea: Cheongju |  | MW081689 | MW467668 |  |  |  |  | This study |
| *Bufo sachalinensis* cf. *sachalinensis* | BGB24 | 36.65 | 127.33 | Republic of Korea: Cheongju |  |  | MW467669 |  |  |  |  | This study |
| *Bufo sachalinensis* cf. *sachalinensis* | BGB110 | 36.67 | 126.64 | Republic of Korea: Chungcheongnam-do |  | MW081690 | MW467670 |  |  |  |  | This study |
| *Bufo sachalinensis* cf. *sachalinensis* | BGB111 | 36.67 | 126.64 | Republic of Korea: Chungcheongnam-do |  | MW081691 | MW467671 |  |  |  |  | This study |
| *Bufo sachalinensis* cf. *sachalinensis* | BGB112 | 36.67 | 126.64 | Republic of Korea: Chungcheongnam-do |  | MW081692 | MW467672 |  |  |  |  | This study |
| *Bufo sachalinensis* cf. *sachalinensis* | BGB113 | 36.67 | 126.64 | Republic of Korea: Chungcheongnam-do |  | MW081693 | MW467673 |  |  |  |  | This study |
| *Bufo sachalinensis* cf. *sachalinensis* | BGB114 | 36.67 | 126.64 | Republic of Korea: Chungcheongnam-do |  | MW081694 | MW467674 |  |  |  |  | This study |
| *Bufo sachalinensis* cf. *sachalinensis* | BGB115 | 36.67 | 126.64 | Republic of Korea: Chungcheongnam-do |  | MW081695 | MW467675 |  |  |  |  | This study |
| *Bufo sachalinensis* cf. *sachalinensis* | BGB116 | 36.67 | 126.64 | Republic of Korea: Chungcheongnam-do |  | MW081696 | MW467676 |  |  |  |  | This study |
| *Bufo sachalinensis* cf. *sachalinensis* | BGB117 | 36.67 | 126.64 | Republic of Korea: Chungcheongnam-do |  | MW081697 | MW467677 |  | MW489990 |  |  | This study |
| *Bufo gargarizans* | BGC1 | 32.18 | 117.14 | China: Hefei |  | MW081698 | MW467678 |  |  |  |  | This study |
| *Bufo gargarizans* | BGC3 | 32.18 | 117.14 | China: Hefei |  |  | MW467679 |  | MW489991 |  |  | This study |
| *Bufo gargarizans* | BGC4 | 32.18 | 117.14 | China: Hefei |  | MW081699 | MW467680 |  |  |  |  | This study |
| *Bufo gargarizans* | BGC017 | 30.7 | 120.78 | China: Jianxing |  | MW081700 |  |  |  |  |  | This study |
| *Bufo gargarizans* | BGC018 | 30.7 | 120.78 | China: Jianxing |  |  |  |  |  |  |  | This study |
| *Bufo sachalinensis* cf. *sachalinensis* | BGD31 | 35.78 | 127.14 | Republic of Korea: Jeollabuk-do |  | MW081701 | MW467681 |  |  |  |  | This study |
| *Bufo sachalinensis* cf. *sachalinensis* | BGD32 | 35.78 | 127.14 | Republic of Korea: Jeollabuk-do |  | MW081702 | MW467682 |  |  |  |  | This study |
| *Bufo sachalinensis* cf. *sachalinensis* | BGD33 | 35.78 | 127.14 | Republic of Korea: Jeollabuk-do |  | MW081703 | MW467683 |  |  |  |  | This study |
| *Bufo sachalinensis* cf. *sachalinensis* | BGD34 | 35.78 | 127.14 | Republic of Korea: Jeollabuk-do |  | MW081704 | MW467684 |  |  |  |  | This study |
| *Bufo sachalinensis* cf. *sachalinensis* | BGD35 | 35.78 | 127.14 | Republic of Korea: Jeollabuk-do |  | MW081705 | MW467685 |  |  |  |  | This study |
| *Bufo sachalinensis* cf. *sachalinensis* | BGD37 | 35.78 | 127.14 | Republic of Korea: Jeollabuk-do |  | MW081707 | MW467686 |  |  |  |  | This study |
| *Bufo sachalinensis* cf. *sachalinensis* | BGD38 | 35.78 | 127.14 | Republic of Korea: Jeollabuk-do |  | MW081708 |  |  |  |  |  | This study |
| *Bufo sachalinensis* cf. *sachalinensis* | BGD39 | 35.78 | 127.14 | Republic of Korea: Jeollabuk-do |  | MW081709 |  | MW489919 |  |  |  | This study |
| *Bufo sachalinensis* cf. *sachalinensis* | BGD41 | 35.62 | 128.4 | Republic of Korea: Gyeongsangnam-do |  | MW081710 |  | MW489920 |  |  |  | This study |
| *Bufo sachalinensis* cf. *sachalinensis* | BGD42 | 35.62 | 128.4 | Republic of Korea: Gyeongsangnam-do |  | MW081711 | MW467687 |  |  |  |  | This study |
| *Bufo sachalinensis* cf. *sachalinensis* | BGD43 | 35.62 | 128.4 | Republic of Korea: Gyeongsangnam-do |  | MW081712 | MW467688 |  |  |  |  | This study |
| *Bufo sachalinensis* cf. *sachalinensis* | BGD44 | 35.62 | 128.4 | Republic of Korea: Gyeongsangnam-do |  | MW081713 | MW467689 |  | MW489992 |  |  | This study |
| *Bufo sachalinensis* cf. *sachalinensis* | BGD45 | 35.62 | 128.4 | Republic of Korea: Gyeongsangnam-do |  | MW081714 | MW467690 | MW489921 | MW489993 | MW507755 |  | This study |
| *Bufo sachalinensis* cf. *sachalinensis* | BGD46 | 35.62 | 128.4 | Republic of Korea: Gyeongsangnam-do |  | MW081715 | MW467691 |  | MW489994 |  |  | This study |
| *Bufo sachalinensis* cf. *sachalinensis* | BGD47 | 35.62 | 128.4 | Republic of Korea: Gyeongsangnam-do |  | MW081716 | MW467692 |  |  |  |  | This study |
| *Bufo sachalinensis* cf. *sachalinensis* | BGD48 | 35.62 | 128.4 | Republic of Korea: Gyeongsangnam-do |  | MW081717 | MW467693 |  |  |  |  | This study |
| *Bufo sachalinensis* cf. *sachalinensis* | BGD49 | 35.62 | 128.4 | Republic of Korea: Gyeongsangnam-do |  | MW081718 | MW467694 |  |  |  |  | This study |
| *Bufo sachalinensis* cf. *sachalinensis* | BGD51 | 35.83 | 128.71 | Republic of Korea: Daegu |  | MW081719 | MW467695 |  |  |  |  | This study |
| *Bufo sachalinensis* cf. *sachalinensis* | BGD52 | 35.83 | 128.71 | Republic of Korea: Daegu |  | MW081720 | MW467696 |  |  |  |  | This study |
| *Bufo sachalinensis* cf. *sachalinensis* | BGD53 | 35.83 | 128.71 | Republic of Korea: Daegu |  | MW081721 | MW467697 |  |  |  |  | This study |
| *Bufo sachalinensis* cf. *sachalinensis* | BGD54 | 35.83 | 128.71 | Republic of Korea: Daegu |  | MW081722 | MW467698 |  | MW489995 |  |  | This study |
| *Bufo sachalinensis* cf. *sachalinensis* | BGD55 | 35.83 | 128.71 | Republic of Korea: Daegu |  | MW081723 | MW467699 |  |  |  |  | This study |
| *Bufo sachalinensis* cf. *sachalinensis* | BGD56 | 35.83 | 128.71 | Republic of Korea: Daegu |  | MW081724 |  |  |  |  |  | This study |
| *Bufo sachalinensis* cf. *sachalinensis* | BGD57 | 35.83 | 128.71 | Republic of Korea: Daegu |  | MW081725 | MW467700 |  |  |  |  | This study |
| *Bufo sachalinensis* cf. *sachalinensis* | BGD58 | 35.83 | 128.71 | Republic of Korea: Daegu |  | MW081726 | MW467701 | MW489922 | MW489996 |  |  | This study |
| *Bufo sachalinensis* cf. *sachalinensis* | BGD310 | 35.78 | 127.14 | Republic of Korea: Jeollabuk-do |  | MW081727 | MW467702 |  |  |  |  | This study |
| *Bufo sachalinensis* cf. *sachalinensis* | BGD311 | 35.78 | 127.14 | Republic of Korea: Jeollabuk-do |  | MW081728 | MW467703 |  |  |  |  | This study |
| *Bufo sachalinensis* cf. *sachalinensis* | BGD312 | 35.78 | 127.14 | Republic of Korea: Jeollabuk-do |  | MW081729 |  | MW489923 |  |  |  | This study |
| *Bufo sachalinensis* cf. *sachalinensis* | BGD313 | 35.78 | 127.14 | Republic of Korea: Jeollabuk-do |  | MW081730 | MW467704 |  |  |  |  | This study |
| *Bufo sachalinensis* cf. *sachalinensis* | BGD314 | 35.78 | 127.14 | Republic of Korea: Jeollabuk-do |  | MW081731 | MW467705 |  |  |  |  | This study |
| *Bufo sachalinensis* cf. *sachalinensis* | BGD410 | 35.62 | 128.4 | Republic of Korea: Gyeongsangnam-do |  |  |  |  | MW489997 |  |  | This study |
| *Bufo sachalinensis* cf. *sachalinensis* | BGD411 | 35.62 | 128.4 | Republic of Korea: Gyeongsangnam-do |  | MW081732 | MW467706 |  |  |  |  | This study |
| *Bufo sachalinensis* cf. *sachalinensis* | BGD412 | 35.62 | 128.4 | Republic of Korea: Gyeongsangnam-do |  | MW081733 | MW467707 |  | MW489998 |  |  | This study |
| *Bufo sachalinensis* cf. *sachalinensis* | BGD413 | 35.62 | 128.4 | Republic of Korea: Gyeongsangnam-do |  | MW081734 | MW467708 |  |  |  |  | This study |
| *Bufo sachalinensis* cf. *sachalinensis* | BGD414 | 35.62 | 128.4 | Republic of Korea: Gyeongsangnam-do |  | MW081735 | MW467709 |  | MW489999 |  |  | This study |
| *Bufo sachalinensis* cf. *sachalinensis* | BGD415 | 35.62 | 128.4 | Republic of Korea: Gyeongsangnam-do |  | MW081736 | MW467710 |  | MW490000 |  |  | This study |
| *Bufo sachalinensis* cf. *sachalinensis* | BGD510 | 35.83 | 128.71 | Republic of Korea: Daegu |  | MW081737 |  |  | MW490001 |  |  | This study |
| *Bufo sachalinensis* cf. *sachalinensis* | BGD511 | 35.83 | 128.71 | Republic of Korea: Daegu |  | MW081738 | MW467711 |  |  |  |  | This study |
| *Bufo sachalinensis* cf. *sachalinensis*. | BGD512 | 35.83 | 128.71 | Republic of Korea: Daegu |  | MW081739 | MW467712 |  | MW490002 |  |  | This study |
| *Bufo sachalinensis* cf. *sachalinensis* | BGD513 | 35.83 | 128.71 | Republic of Korea: Daegu |  | MW081740 | MW467713 |  | MW490003 |  |  | This study |
| *Bufo sachalinensis* cf. *sachalinensis*. | BGD514 | 35.83 | 128.71 | Republic of Korea: Daegu |  | MW081741 | MW467714 |  |  |  |  | This study |
| *Bufo sachalinensis* cf. *sachalinensis* | BGD515 | 35.83 | 128.71 | Republic of Korea: Daegu |  | MW081742 | MW467715 | MW489924 | MW490004 | MW507756 |  | This study |
| *Bufo sachalinensis* cf. *sachalinensis* | BGD516 | 35.83 | 128.71 | Republic of Korea: Daegu |  | MW081743 |  |  | MW490005 |  |  | This study |
| *Bufo sachalinensis* cf. *sachalinensis* | BGD517 | 35.83 | 128.71 | Republic of Korea: Daegu |  | MW081744 | MW467716 | MW489925 |  |  |  | This study |
| *Bufo sachalinensis* cf. *sachalinensis* | BGD518 | 35.83 | 128.71 | Republic of Korea: Daegu |  | MW081745 |  |  |  |  |  | This study |
| *Bufo sachalinensis* cf. *sachalinensis* | BGD519 | 35.83 | 128.71 | Republic of Korea: Daegu |  | MW081746 | MW467717 |  |  |  |  | This study |
| *Bufo sachalinensis* cf. *sachalinensis* | BGD520 | 35.83 | 128.71 | Republic of Korea: Daegu |  | MW081747 | MW467718 |  |  |  |  | This study |
| *Bufo sachalinensis* cf. *sachalinensis* | BGD521 | 35.83 | 128.71 | Republic of Korea: Daegu |  | MW081748 |  |  |  |  |  | This study |
| *Bufo sachalinensis* cf. *sachalinensis* | BGD522 | 35.83 | 128.71 | Republic of Korea: Daegu |  | MW081749 | MW467719 |  |  |  |  | This study |
| *Bufo sachalinensis* cf. *sachalinensis* | BGD523 | 35.83 | 128.71 | Republic of Korea: Daegu |  | MW081750 | MW467720 |  |  |  |  | This study |
| *Bufo sachalinensis* cf. *sachalinensis* | BGD524 | 35.83 | 128.71 | Republic of Korea: Daegu |  | MW081751 |  | MW489926 | MW490006 | MW507757 |  | This study |
| *Bufo sachalinensis* cf. *sachalinensis* | BGD525 | 35.83 | 128.71 | Republic of Korea: Daegu |  | MW081752 | MW467721 |  | MW490007 |  |  | This study |
| *Bufo sachalinensis* cf. *sachalinensis* | BGE25 | 35.15 | 127.67 | Republic of Korea: Gyeongsangnam-do |  | MW081753 |  |  |  |  |  | This study |
| *Bufo sachalinensis* cf. *sachalinensis* | BGE31 | 35.15 | 127.67 | Republic of Korea: Gyeongsangnam-do |  | MW081754 | MW467722 | MW489927 | MW490008 | MW507758 |  | This study |
| *Bufo sachalinensis* cf. *sachalinensis* | BGE32 | 35.15 | 127.67 | Republic of Korea: Gyeongsangnam-do |  | MW081755 |  |  |  |  |  | This study |
| *Bufo sachalinensis* cf. *sachalinensis* | BGE33 | 35.15 | 127.67 | Republic of Korea: Gyeongsangnam-do |  | MW081756 |  |  | MW490009 |  |  | This study |
| *Bufo sachalinensis* cf. *sachalinensis* | BGE34 | 35.15 | 127.67 | Republic of Korea: Gyeongsangnam-do |  | MW081757 | MW467723 |  |  |  |  | This study |
| *Bufo sachalinensis* cf. *sachalinensis* | BGE51 | 35.29 | 128.67 | Republic of Korea: Changwon-si, Gyeongsangnam-do |  | MW081758 |  |  | MW490010 |  |  | This study |
| *Bufo sachalinensis* cf. *sachalinensis* | BGE52 | 35.29 | 128.67 | Republic of Korea: Changwon-si, Gyeongsangnam-do |  | MW081759 | MW467724 |  | MW490011 |  |  | This study |
| *Bufo sachalinensis* cf. *sachalinensis* | BGE53 | 35.29 | 128.67 | Republic of Korea: Changwon-si, Gyeongsangnam-do |  | MW081760 | MW467725 |  |  |  |  | This study |
| *Bufo sachalinensis* cf. *sachalinensis* | BGE54 | 35.29 | 128.67 | Republic of Korea: Changwon-si, Gyeongsangnam-do |  | MW081761 | MW467726 |  |  |  |  | This study |
| *Bufo gargarizans* | BR2 | 34.29 | 110.05 | China: Rong Hua Shan Zhen |  | MW081762 | MW467727 |  |  |  |  | This study |
| *Bufo s. sachalinensis* | BR2N | 47.02 | 142.43 | Russia: Novoaleksandrovsk, Sakhalin |  | MW081763 |  |  |  |  |  | This study |
| *Bufo s. sachalinensis* | BR11 | 47.02 | 142.43 | Russia: Novoaleksandrovsk, Sakhalin |  | MW081764 |  | MW489928 |  |  |  | This study |
| *Bufo s. sachalinensis* | BR12 | 47.16 | 142.08 | Russia: Pioner, Sakhalin |  | MW081765 |  | MW489929 | MW490012 | MW507759 |  | This study |
| *Bufo s. sachalinensis* | BR21 | 47.18 | 141.58 | Russia: Slepkovskogo Cape, Sakhalin |  |  | MW467728 |  |  |  |  | This study |
| *Bufo gargarizans* | BR64 |  |  | China |  | MW081766 |  |  |  |  |  | This study |
| *Bufo gargarizans* | BR90 | 30.91 | 103.56 | China: Quinchengshan |  | MW081767 | MW467729 |  |  |  |  | This study |
| *Bufo gargarizans* | BR92 | 30.91 | 103.56 | China: Quinchengshan |  |  | MW467730 |  |  |  |  | This study |
| *Bufo andrewsi* | BR121 | 29.52 | 103.33 | China: Emei Mt. |  | MW081768 |  | MW489930 | MW490013 | MW507760 |  | This study |
| *Bufo gargarizans* | BR178 |  |  | China |  | MW081769 | MW467731 |  |  |  |  | This study |
| *Bufo gargarizans* | BR317 |  |  | China |  | MW081770 |  |  |  |  |  | This study |
| *Bufo gargarizans* | BR505 | 38.91 | 121.63 | China: Dalian, park Lao Dong |  | MW081771 | MW467732 | MW489931 | MW490014 | MW507761 |  | This study |
| *Bufo gargarizans* | BR517 | 40.22 | 124.5 | China: Hushancun Ai River |  | MW081772 | MW467733 | MW489932 | MW490015 | MW507762 |  | This study |
| *Bufo gargarizans* | BR518 | 38.93 | 121.49 | China: Dalian, Daxishan Reservoir |  | MW081773 |  | MW489933 |  | MW507763 |  | This study |
| *Bufo gargarizans* | BR519 | 38.93 | 121.49 | China: Dalian, Daxishan Reservoir |  | MW081774 |  |  |  |  |  | This study |
| *Bufo gargarizans* | BR520 | 38.93 | 121.49 | China: Dalian, Daxishan Reservoir |  | MW081775 | MW467734 |  |  |  |  | This study |
| *Bufo gargarizans* | BR522 | 38.93 | 121.49 | China: Dalian, Daxishan Reservoir |  | MW081776 |  |  |  |  |  | This study |
| *Bufo s. sachalinensis* | BR529 | 42.43 | 130.47 | Russia: Kraskino, Primosky |  | MW081777 |  |  |  |  |  | This study |
| *Bufo gargarizans* | BR552 | 39.95 | 122.87 | China: Rong Hua Shan Zhen |  | MW081778 | MW467735 | MW489934 |  |  |  | This study |
| *Bufo gargarizans* | BR554 | 39.95 | 122.87 | China: Rong Hua Shan Zhen |  | MW081779 | MW467736 |  |  |  |  | This study |
| *Bufo gargarizans* | BR565 | 39.95 | 122.87 | China: Rong Hua Shan Zhen |  | MW081780 | MW467737 | MW489935 |  |  |  | This study |
| *Bufo gargarizans* | BR566 | 38.91 | 121.63 | China: Dalian, park Lao Dong |  | MW081781 | MW467738 | MW489936 |  |  |  | This study |
| *Bufo gargarizans* | BR568 | 38.91 | 121.63 | China: Dalian, park Lao Dong |  | MW081782 | MW467739 | MW489937 | MW490016 | MW507764 |  | This study |
| *Bufo gargarizans* | BR575 | 33.85 | 107.82 | China: Heuzhenzizhen |  | MW081783 |  | MW489938 |  |  |  | This study |
| *Bufo s. sachalinensis* | BR589 | 43.49 | 142.46 | Russia: Antonikha River, Sakhalin |  | MW081784 | MW467740 |  |  |  |  | This study |
| *Bufo s. sachalinensis* | BR630 |  |  | China |  | MW081785 | MW467741 |  |  |  |  | This study |
| *Bufo andrewsi* | BR651 | 33.85 | 107.82 | China: Heuzhenzizhen |  | MW081786 |  | MW489939 | MW490017 | MW507765 |  | This study |
| *Bufo andrewsi* | BR652 | 33.85 | 107.82 | China: Heuzhenzizhen |  | MW081787 | MW467742 | MW489940 | MW490018 | MW507766 |  | This study |
| *Bufo andrewsi* | BR653 | 33.85 | 107.82 | China: Heuzhenzizhen |  | MW081788 |  | MW489941 | MW490019 | MW507767 |  | This study |
| *Bufo andrewsi* | BR654 | 33.85 | 107.82 | China: Heuzhenzizhen |  | MW081789 |  |  | MW490020 |  |  | This study |
| *Bufo andrewsi* | BR655 | 33.85 | 107.82 | China: Heuzhenzizhen |  | MW081790 |  | MW489942 | MW490021 |  |  | This study |
| *Bufo andrewsi* | BR666 | 33.85 | 107.82 | China: Heuzhenzizhen |  | MW081791 | MW467743 |  | MW490022 |  |  | This study |
| *Bufo andrewsi* | BR667 | 33.85 | 107.82 | China: Heuzhenzizhen |  |  |  | MW489943 | MW490023 | MW507768 |  | This study |
| *Bufo andrewsi* | BR668 | 33.85 | 107.82 | China: Heuzhenzizhen |  | MW081792 |  | MW489944 |  |  |  | This study |
| *Bufo gargarizans* | BR669 | 29.35 | 103.2 | China: Wan-nian-cun |  | MW081793 | MW467744 |  |  |  |  | This study |
| *Bufo gargarizans* | BR670 | 29.35 | 103.2 | China: Wan-nian-cun |  | MW081794 | MW467745 | MW489945 |  |  |  | This study |
| *Bufo gargarizans* | BR671 | 29.35 | 103.2 | China: Wan-nian-cun |  | MW081795 | MW467746 |  |  |  |  | This study |
| *Bufo gargarizans* | BR672 | 29.35 | 103.2 | China: Wan-nian-cun |  | MW081796 | MW467747 | MW489946 | MW490024 |  |  | This study |
| *Bufo s. sachalinensis* | BR676 | 43.43 | 132.02 | Russia: Bonevurovka, Primorsky |  | MW081797 |  |  |  |  |  | This study |
| *Bufo s. sachalinensis* | BR679 | 43.43 | 132.02 | Russia: Bonevurovka, Primorsky |  | MW081798 |  |  |  |  |  | This study |
| *Bufo s. sachalinensis* | BR682 | 43.43 | 132.02 | Russia: Bonevurovka, Primorsky |  | MW081799 | MW467748 |  |  |  |  | This study |
| *Bufo s. sachalinensis* | BR689 | 43.43 | 132.02 | Russia: Bonevurovka, Primorsky |  | MW081800 | MW467749 |  |  |  |  | This study |
| *Bufo s. sachalinensis* | BR690 | 43.43 | 132.02 | Russia: Bonevurovka, Primorsky |  | MW081801 | MW467750 |  |  |  |  | This study |
| *Bufo s. sachalinensis* | BR691 | 43.43 | 132.02 | Russia: Bonevurovka, Primorsky |  | MW081802 | MW467751 |  |  |  |  | This study |
| *Bufo s. sachalinensis* | BR692 | 43.43 | 132.02 | Russia: Bonevurovka, Primorsky |  | MW081803 | MW467752 |  |  |  |  | This study |
| *Bufo s. sachalinensis* | BR693 | 43.43 | 132.02 | Russia: Bonevurovka, Primorsky |  | MW081804 | MW467753 |  |  |  |  | This study |
| *Bufo s. sachalinensis* | BR694 | 43.43 | 132.02 | Russia: Bonevurovka, Primorsky |  | MW081805 | MW467754 |  |  |  |  | This study |
| *Bufo s. sachalinensis* | BR695 | 43.43 | 132.02 | Russia: Bonevurovka, Primorsky |  | MW081806 | MW467755 |  |  |  |  | This study |
| *Bufo s. sachalinensis* | BR698 | 43.43 | 132.02 | Russia: Bonevurovka, Primorsky |  | MW081807 |  |  |  |  |  | This study |
| *Bufo s. sachalinensis* | BR699 | 43.43 | 132.02 | Russia: Bonevurovka, Primorsky |  | MW081808 | MW467756 | MW489947 |  |  |  | This study |
| *Bufo s. sachalinensis* | BR702 | 47.11 | 142.27 | Russia: Sinegorsk, Sakhalin |  | MW081809 | MW467757 | MW489948 |  |  |  | This study |
| *Bufo gargarizans* | BR708 | 29.35 | 103.2 | China: Wan-nian-cun |  | MW081810 | MW467758 | MW489949 |  |  |  | This study |
| *Bufo andrewsi* | BR710 | 33.85 | 107.82 | China: Heuzhenzizhen |  | MW081811 | MW467759 | MW489950 | MW490025 | MW507769 |  | This study |
| *Bufo andrewsi* | BR711 | 33.85 | 107.82 | China: Heuzhenzizhen |  | MW081812 | MW467760 |  |  |  |  | This study |
| *Bufo andrewsi* | BR712 | 33.85 | 107.82 | China: Heuzhenzizhen |  | MW081813 |  | MW489951 | MW490026 | MW507770 |  | This study |
| *Bufo gargarizans* | BR725 |  |  | China |  | MW081814 |  |  |  |  |  | This study |
| *Bufo s. sachalinensis* | BR793 | 42.56 | 132.23 | Russia: Krym |  | MW081815 |  | MW489952 |  |  |  | This study |
| *Bufo s. sachalinensis* | BR794 | 42.27 | 130.38 | Russia: Khasan, Primorsky |  | MW081816 | MW467761 | MW489953 | MW490027 | MW507771 |  | This study |
| *Bufo s. sachalinensis* | BR795 | 42.43 | 130.64 | Russia: Khasan, Primorsky |  | MW081817 | MW467762 | MW489954 |  |  |  | This study |
| *Bufo s. sachalinensis* | BR876 | 47.17 | 142.31 | Russia: Sinegorsk, Sakhalin |  | MW081818 |  |  |  |  |  | This study |
| *Bufo s. sachalinensis* | BR877 | 47.17 | 142.31 | Russia: Sinegorsk, Sakhalin |  | MW081819 |  |  |  |  |  | This study |
| *Bufo s. sachalinensis* | BR878 | 47.17 | 142.31 | Russia: Sinegorsk, Sakhalin |  | MW081820 | MW467763 |  |  |  |  | This study |
| *Bufo s. sachalinensis* | BR879 | 47.17 | 142.31 | Russia: Sinegorsk, Sakhalin |  | MW081821 | MW467764 |  |  |  |  | This study |
| *Bufo s. sachalinensis* | BR881 | 42.56 | 132.23 | Russia: Krym, Primorsky |  | MW081822 |  |  |  |  |  | This study |
| *Bufo s. sachalinensis* | BR882 | 42.56 | 132.23 | Russia: Krym, Primorsky |  | MW081823 | MW467765 |  |  |  |  | This study |
| *Bufo s. sachalinensis* | BR884 | 42.56 | 132.23 | Russia: Krym, Primorsky |  | MW081824 | MW467766 |  |  |  |  | This study |
| *Bufo s. sachalinensis* | BR964 | 46.42 | 141.55 | Russia: Pridorozhnyi, Sakhalin |  | MW081825 |  |  |  |  |  | This study |
| *Bufo s. sachalinensis* | BR965 | 46.42 | 141.55 | Russia: Pridorozhnyi, Sakhalin |  | MW081826 |  |  |  |  |  | This study |
| *Bufo s. sachalinensis* | BR966 | 46.42 | 141.55 | Russia: Pridorozhnyi, Sakhalin |  | MW081827 |  |  |  |  |  | This study |
| *Bufo s. sachalinensis* | BR968 | 46.42 | 141.55 | Russia: Pridorozhnyi, Sakhalin |  | MW081828 | MW467767 |  |  |  |  | This study |
| *Bufo s. sachalinensis* | BR969 | 46.42 | 141.55 | Russia: Pridorozhnyi, Sakhalin |  | MW081829 | MW467768 |  |  |  |  | This study |
| *Bufo s. sachalinensis* | BR972 | 47.04 | 142.05 | Russia: Kholmsk, Sakhalin |  | MW081830 | MW467769 |  |  |  |  | This study |
| *Bufo s. sachalinensis* | BR983 | 47.04 | 142.05 | Russia: Kholmsk, Sakhalin |  | MW081831 | MW467770 |  |  |  |  | This study |
| *Bufo gargarizans* | CBg001Jinlong | 31.91 | 118.81 | China: Jinlong Park, Jiangsu, Nanjing |  | MW081832 | MW467771 |  |  |  |  | This study |
| *Bufo gargarizans* | CBg002Jinlong | 31.81 | 118.87 | China: Jinlong Park, Jiangsu, Nanjing |  | MW081833 |  |  |  |  |  | This study |
| *Bufo gargarizans* | CBg003 | 32.03 | 118.84 | China: Jinlong Park, Jiangsu, Nanjing |  |  | MW467772 |  |  |  |  | This study |
| *Bufo gargarizans* | CBg007 | 31.91 | 118.81 | China: Jiangsu, Nanjing |  | MW081834 | MW467773 |  |  |  |  | This study |
| *Bufo gargarizans* | CBgZNP2 | 32.07 | 118.83 | China: Zhanjung Shan |  | MW081835 |  |  |  |  |  | This study |
| *Bufo gargarizans* | SHS1B1 | 31.05 | 121.04 | China: Shanghai |  | MW081848 | MW467778 |  |  |  |  | This study |
| *Bufo gargarizans* | SHS1B2 | 31.05 | 121.04 | China: Shanghai |  | MW081849 |  |  |  |  |  | This study |
| *Bufo gargarizans* | SHS1B4 | 31.05 | 121.04 | China: Shanghai |  | MW081850 | MW467779 |  | MW490036 |  |  | This study |
| *Bufo gargarizans* | SHS2B7 | 31.05 | 121.04 | China: Shanghai |  | MW081851 | MW467780 | MW489965 |  |  |  | This study |
| *Bufo gargarizans* | SHS7B13 | 31.2 | 121.04 | China: Shanghai |  | MW081852 | MW467781 | MW489966 | MW490037 | MW507781 |  | This study |
| *Bufo gargarizans* | CLPT317 | 32.066 | 118.836 | China: Purple mountain cave mouth, Nanjing |  | MW081836 | MW467774 | MW489955 | MW490028 | MW507772 |  | This study |
| *Bufo gargarizans* | CLPT320 | 32.066 | 118.836 | China: Purple mountain cave mouth, Nanjing |  | MW081837 | MW467775 | MW489956 |  | MW507773 |  | This study |
| *Bufo gargarizans* | CLPT325 | 32.76 | 109.09 | China: Anzihe Preserve, Sichuan |  | MW081838 |  | MW489957 | MW490029 | MW507774 |  | This study |
| *Bufo gargarizans* | CLPT326 | 32.76 | 109.09 | China: Anzihe Preserve, Sichuan |  | MW081839 |  |  |  |  |  | This study |
| *Bufo gargarizans* | CLPT327 | 32.76 | 109.09 | China: Anzihe Preserve, Sichuan |  | MW081840 |  |  |  |  |  | This study |
| *Bufo gargarizans* | CLPT331 | 27.73 | 118.046 | Wuyishan, Fujian |  |  | MW467776 | MW489958 | MW490030 | MW507775 |  | This study |
| *Bufo gargarizans* | CLPT349 | 31.76 | 110.68 | China: Shennongjia-Guanmenshan, Hefei |  | MW081841 |  |  | MW490031 |  |  | This study |
| *Bufo gargarizans* | CLPT351 | 31.76 | 110.68 | China: Shennongjia-Guanmenshan, Hefei |  | MW081842 |  | MW489959 | MW490032 | MW507776 |  | This study |
| *Bufo gargarizans* | CLPT364 | 31.76 | 110.68 | China: Shennongjia-Guanmenshan, Hefei |  | MW081843 |  | MW489960 | MW490033 | MW507777 |  | This study |
| *Bufo gargarizans* | CLPT398 | 31.76 | 110.68 | China: Shennongjia-Guanmenshan, Hefei |  | MW081844 |  | MW489961 | MW490034 | MW507778 |  | This study |
| *Bufo gargarizans* | CLPT399 | 31.76 | 110.68 | China: Shennongjia-Guanmenshan, Hefei |  |  |  |  |  |  |  | This study |
| *Bufo gargarizans* | CLPT403 | 31.72 | 110.68 | China: Shennongjia, Dongxi |  | MW081845 |  | MW489962 | MW490035 | MW507779 |  | This study |
| *Bufo gargarizans* | CLPT404 | 31.72 | 110.68 | China: Shennongjia, Dongxi |  | MW081846 |  | MW489963 |  |  |  | This study |
| *Bufo gargarizans* | CLPT407 | 31.72 | 110.68 | China: Shennongjia, Dongxi |  | MW081847 | MW467777 | MW489964 |  | MW507780 |  | This study |
| *Bufo gargarizans “minshanicus”* |  |  |  | China: Danba County, Sichuan Province |  | KM587710 | KM587710 |  |  |  |  | Yang et al. (2015) |
| *Bufo tibetanus* | KIZ-97L004 | 27.7 | 99.7 | China: Zhongdian |  | AF190249 | AY936839 |  |  |  |  | Fu et al. (2005) |
| *Bufo tibetanus* | KIZ-97001 | 27.7 | 99.7 | China: Zhongdian |  | AY924309 | AY936838 |  |  |  |  | Fu et al. (2005) |
| *Bufo andrewsi* | CIBJF082 | 30.3 | 102.8 | China: Baoxing |  | AY924319 | AY936844 |  |  |  |  | Fu et al. (2005) |
| *Bufo andrewsi* | CIBJF098 | 30.01 | 101.28 | China: Xinduqiao |  | AY924368 | AY936861 |  |  |  |  | Fu et al. (2005) |
| *Bufo gargarizans* | CIBJF231 | 30.2 | 119.7 | China: Lin’an |  | AY924348 | AY936853 |  |  |  |  | Fu et al. (2005) |
| *Bufo gargarizans* | CIBJF232 | 30.2 | 119.7 | China: Lin’an |  | AY924349 | AY936854 |  |  |  |  | Fu et al. (2005) |
| *Bufo gargarizans* | CIBJF252 | 27.43 | 117.39 | China: Guadun |  | AY924350 | AY936874 |  |  |  |  | Fu et al. (2005) |
| *Bufo andrewsi* | CIBJF382 | 31.24 | 100.37 | China: Luhou |  | AY924369 | AY936862 |  |  |  |  | Fu et al. (2005) |
| *Bufo andrewsi* | CIBJF643 | 33.03 | 104.41 | China: Wengxian |  | AY924340 | AY936849 |  |  |  |  | Fu et al. (2005) |
| *Bufo andrewsi* | CIBJF654 | 33.03 | 104.41 | China: Wengxian |  | AY924342 | AY936851 |  |  |  |  | Fu et al. (2005) |
| *Bufo gargarizans* | CIBW01411 | 39.47 | 115.24 | China: Baihuashan |  | AY924365 | AY936859 |  |  |  |  | Fu et al. (2005) |
| *Bufo gargarizans* | CIBW01412 | 39.47 | 115.24 | China: Baihuashan |  | AY924364 | AY936863 |  |  |  |  | Fu et al. (2005) |
| *Bufo gargarizans* | CIBXM014 | 42.32 | 128.18 | China: Antu |  | AY924344 | AY936870 |  |  |  |  | Fu et al. (2005) |
| *Bufo gargarizans* | CIBXM076 | 42.32 | 128.18 | China: Antu |  | AY924345 | AY936867 |  |  |  |  | Fu et al. (2005) |
| *Bufo andrewsi* | CIBXM086 | 31 | 104.6 | China: Zhongjiang |  | AY924352 | AY936855 |  |  |  |  | Fu et al. (2005) |
| *Bufo andrewsi* | CIBXM087 | 31 | 104.6 | China: Zhongjiang |  | AY924353 | AY936856 |  |  |  |  | Fu et al. (2005) |
| *Bufo andrewsi* | CIBXM099 | 31.14 | 103.45 | China: Pengxian |  | AY924361 | AY936868 |  |  |  |  | Fu et al. (2005) |
| *Bufo andrewsi* | CIBXM128 | 29.35 | 103.17 | China: Omei Mt.-II |  | AY924321 | AY936869 |  |  |  |  | Fu et al. (2005) |
| *Bufo andrewsi* | CIBXM281 | 29.39 | 102.57 | China: Hongya |  | AY924354 | AY936857 |  |  |  |  | Fu et al. (2005) |
| *Bufo andrewsi* | CIBXM557 | 28.5 | 102.1 | China: Mianning |  | AY924315 | AY936842 |  |  |  |  | Fu et al. (2005) |
| *Bufo andrewsi* | CIBXM558 | 28.5 | 102.1 | China: Mianning |  | AY924316 | AY936843 |  |  |  |  | Fu et al. (2005) |
| *Bufo andrewsi* | CIBXM612 | 28.7 | 99.2 | China: Derong |  | AY924311 | AY936865 |  |  |  |  | Fu et al. (2005) |
| *Bufo andrewsi* | CIBZYC012 | 30.14 | 103.05 | China: Qionglai |  | AY924330 | AY936846 |  |  |  |  | Fu et al. (2005) |
| *Bufo andrewsi* | CIBZYC013 | 30.14 | 103.05 | China: Qionglai |  | AY924329 | AY936845 |  |  |  |  | Fu et al. (2005) |
| *Bufo andrewsi* | CIBZYC061 | 31.41 | 103.52 | China: Maoxian |  | AY924333 | AY936847 |  |  |  |  | Fu et al. (2005) |
| *Bufo andrewsi* | CIBZYC062 | 31.41 | 103.52 | China: Maoxian |  | AY924334 | AY936848 |  |  |  |  | Fu et al. (2005) |
| *Bufo gargarizans* | CIBZYC579 | 29.3 | 110.4 | China: Zhangjiajie |  | AY924346 | AY936871 |  |  |  |  | Fu et al. (2005) |
| *Bufo gargarizans* | CIBZYC668 | 27.53 | 108.42 | China: Jiangkou |  | AY924343 | AY936852 |  |  |  |  | Fu et al. (2005) |
| *Bufo gargarizans* | CIBZYC782 | 29.04 | 107.11 | China: Nanchuan |  | AY924351 | AY936854 |  |  |  |  | Fu et al. (2005) |
| *Bufo andrewsi* |  |  |  | China |  |  | AF004529 |  |  |  |  | Macey et al. (1998) |
| *Bufo andrewsi* | CIBZYC907 | 32.03 | 108.1 | China: Wangyuan |  | AY924338 | AY936876 |  |  |  |  | Fu et al. (2005) |
| *Bufo andrewsi* | KIZ95L004 | 25 | 102.7 | China: Kunming |  | AF190230 | AY936866 |  |  |  |  | Fu et al. (2005) |
| *Bufo andrewsi* | KIZ97005 | 27.7 | 99.7 | China: Zhongdian |  | AY924310 | AY936840 |  |  |  |  | Fu et al. (2005) |
| *Bufo andrewsi* | KIZ97006 | 25 | 102.7 | China: Kunming |  | AY924312 | AY936841 |  |  |  |  | Fu et al. (2005) |
| *Bufo andrewsi* | UG37431 | 29.35 | 103.17 | China: Omei Mt. |  | AY924356 | AY936858 |  |  |  |  | Fu et al. (2005) |
| *Bufo gargarizans* | UG37763 | 39.47 | 115.24 | China: Baihuashan |  | AY924364 | AY936863 |  |  |  |  | Fu et al. (2005) |
| *Bufo sachalinensis* cf. *sachalinensis* | Bufo gargarizans 1 | 37.72 | 126.4 | Republic of Korea |  | KY295993 | KY295992 |  |  |  |  | Borzée et al. (2017) |
| *Bufo sachalinensis* cf. *sachalinensis* | Bufo gargarizans 2 | 37.72 | 126.4 | Republic of Korea |  | KY295994 | KY295991 |  |  |  |  | Borzée et al. (2017) |
| *Bufo sachalinensis* cf. *sachalinensis* | Bufo gargarizans 3 | 35.78 | 127.14 | Republic of Korea |  | KY295995 | KY295990 |  |  |  |  | Borzée et al. (2017) |
| *Bufo sachalinensis* cf. *sachalinensis* | Bufo gargarizans 4 | 35.78 | 127.14 | Republic of Korea |  | KY295996 | KY295989 |  |  |  |  | Borzée et al. (2017) |
| *Bufo sachalinensis* cf. *sachalinensis* | Bufo gargarizans 5 | 35.78 | 127.14 | Republic of Korea |  | KY295997 | KY295988 |  |  |  |  | Borzée et al. (2017) |
| *Bufo sachalinensis* cf. *sachalinensis* | Bufo gargarizans 6 | 35.78 | 127.14 | Republic of Korea |  | KY295998 | KY295987 |  |  |  |  | Borzée et al. (2017) |
| *Bufo sachalinensis* cf. *sachalinensis* | Bufo gargarizans 7 | 35.78 | 127.14 | Republic of Korea |  | KY295999 | KY295986 |  |  |  |  | Borzée et al. (2017) |
| *Bufo sachalinensis* cf. *sachalinensis* | Bufo gargarizans 8 | 36.14 | 127.38 | Republic of Korea |  | KY296000 | KY295985 |  |  |  |  | Borzée et al. (2017) |
| *Bufo sachalinensis* cf. *sachalinensis* | Bufo gargarizans 9 | 36.14 | 127.38 | Republic of Korea |  | KY296001 | KY295984 |  |  |  |  | Borzée et al. (2017) |
| *Bufo sachalinensis* cf. *sachalinensis* | Bufo gargarizans 10 | 36.14 | 127.38 | Republic of Korea |  | KY296002 | KY295983 |  |  |  |  | Borzée et al. (2017) |
| *Bufo sachalinensis* cf. *sachalinensis* | Bufo gargarizans 11 | 36.14 | 127.38 | Republic of Korea |  | KY296003 | KY295982 |  |  |  |  | Borzee et al., (2016) |
| *Bufo sachalinensis* cf. *sachalinensis* | Bufo gargarizans 12 | 36.14 | 127.38 | Republic of Korea |  | KY296004 | KY295981 |  |  |  |  | Borzée et al. (2017) |
| *Bufo sachalinensis* cf. *sachalinensis* | Bufo gargarizans 13 | 36.14 | 127.38 | Republic of Korea |  | KY296005 | KY295980 |  |  |  |  | Borzée et al. (2017) |
| *Bufo sachalinensis* cf. *sachalinensis* | Bufo gargarizans 14 | 36.14 | 127.38 | Republic of Korea |  | KY296006 | KY295979 |  |  |  |  | Borzée et al. (2017) |
| *Bufo sachalinensis* cf. *sachalinensis* | Bufo gargarizans 15 | 35.83 | 128.7 | Republic of Korea |  | KY296007 | KY295978 |  |  |  |  | Borzée et al. (2017) |
| *Bufo sachalinensis* cf. *sachalinensis* | Bufo gargarizans 16 | 35.83 | 128.7 | Republic of Korea |  | KY296008 | KY295977 |  |  |  |  | Borzée et al. (2017) |
| *Bufo sachalinensis* cf. *sachalinensis* | Bufo gargarizans 17 | 35.83 | 128.7 | Republic of Korea |  | KY296009 | KY295971 |  |  |  |  | Borzée et al. (2017) |
| *Bufo sachalinensis* cf. *sachalinensis* | Bufo gargarizans 18 | 35.83 | 128.7 | Republic of Korea |  | KY296010 | KY295976 |  |  |  |  | Borzée et al. (2017) |
| *Bufo sachalinensis* cf. *sachalinensis* | Bufo gargarizans 20 | 35.83 | 128.7 | Republic of Korea |  | KY296012 | KY295974 |  |  |  |  | Borzée et al. (2017) |
| *Bufo sachalinensis* cf. *sachalinensis* | Bufo gargarizans 21 | 35.83 | 128.7 | Republic of Korea |  | KY296013 | KY295973 |  |  |  |  | Borzée et al. (2017) |
| *Bufo sachalinensis* cf. *sachalinensis* | Bufo gargarizans 22 | 36.48 | 127.73 | Republic of Korea |  | KY296014 | KY295972 |  |  |  |  | Borzée et al. (2017) |
| *Bufo sachalinensis* cf. *sachalinensis* | Bufo gargarizans 23 | 36.48 | 127.73 | Republic of Korea |  | KY296015 | KY295970 |  |  |  |  | Borzée et al. (2017) |
| *Bufo sachalinensis* cf. *sachalinensis* | Bufo gargarizans 24 | 36.48 | 127.73 | Republic of Korea |  | KY296017 | KY295969 |  |  |  |  | Borzée et al. (2017) |
| *Bufo sachalinensis* cf. *sachalinensis* | Bufo gargarizans 25 | 35.18 | 126.54 | Republic of Korea |  | KY296018 | KY295968 |  |  |  |  | Borzée et al. (2017) |
| *Bufo sachalinensis* cf. *sachalinensis* | Bufo gargarizans 26 | 35.18 | 126.54 | Republic of Korea |  | KY296019 | KY295967 |  |  |  |  | Borzée et al. (2017) |
| *Bufo sachalinensis* cf. *sachalinensis* | Bufo gargarizans 27 | 35.18 | 126.54 | Republic of Korea |  | KY296020 | KY295966 |  |  |  |  | Borzée et al. (2017) |
| *Bufo sachalinensis* cf. *sachalinensis* | Bufo gargarizans 28 | 35.18 | 126.54 | Republic of Korea |  | KY296021 | KY295965 |  |  |  |  | Borzée et al. (2017) |
| *Bufo sachalinensis* cf. *sachalinensis* | Bufo gargarizans 29 | 35.18 | 126.54 | Republic of Korea |  | KY296016 | KY295964 |  |  |  |  | Borzée et al. (2017) |
| *Bufo sachalinensis* cf. *sachalinensis* | Bufo gargarizans 30 | 36.67 | 126.64 | Republic of Korea |  | KY296022 | KY295963 |  |  |  |  | Borzée et al. (2017) |
| *Bufo sachalinensis* cf. *sachalinensis* | Bufo gargarizans 31 | 36.67 | 126.64 | Republic of Korea |  | KY296023 | KY295962 |  |  |  |  | Borzée et al. (2017) |
| *Bufo sachalinensis* cf. *sachalinensis* | Bufo gargarizans 32 | 36.67 | 126.64 | Republic of Korea |  | KY296024 | KY295961 |  |  |  |  | Borzée et al. (2017) |
| *Bufo sachalinensis* cf. *sachalinensis* | Bufo gargarizans 33 | 36.67 | 126.64 | Republic of Korea |  | KY296025 | KY295959 |  |  |  |  | Borzée et al. (2017) |
| *Bufo sachalinensis* cf. *sachalinensis* | Bufo gargarizans 35 | 36.08 | 127.49 | Republic of Korea |  | KY296027 | KY295958 |  |  |  |  | Borzée et al. (2017) |
| *Bufo sachalinensis* cf. *sachalinensis* | Bufo gargarizans 36 | 36.08 | 127.49 | Republic of Korea |  | KY296028 | KY295957 |  |  |  |  | Borzée et al. (2017) |
| *Bufo sachalinensis* cf. *sachalinensis* | Bufo gargarizans 37 | 36.08 | 127.49 | Republic of Korea |  | KY296029 | KY295956 |  |  |  |  | Borzée et al. (2017) |
| *Bufo sachalinensis* cf. *sachalinensis* | Bufo gargarizans 38 | 36.08 | 127.49 | Republic of Korea |  | KY296030 | KY295955 |  |  |  |  | Borzée et al. (2017) |
| *Bufo sachalinensis* cf. *sachalinensis* | Bufo gargarizans 39 | 36.08 | 127.49 | Republic of Korea |  | KY296031 | KY295954 |  |  |  |  | Borzée et al. (2017) |
| *Bufo sachalinensis* cf. *sachalinensis* | Bufo gargarizans 40 | 36.08 | 127.49 | Republic of Korea |  | KY296032 | KY295953 |  |  |  |  | Borzée et al. (2017) |
| *Bufo sachalinensis* cf. *sachalinensis* | Bufo gargarizans 41 | 36.08 | 127.49 | Republic of Korea |  | KY296033 | KY295952 |  |  |  |  | Borzée et al. (2017) |
| *Bufo sachalinensis* cf. *sachalinensis* | Bufo gargarizans 43 | 36.08 | 127.49 | Republic of Korea |  | KY296035 | KY295950 |  |  |  |  | Borzée et al. (2017) |
| *Bufo sachalinensis* cf. *sachalinensis* | Bufo gargarizans 44 | 36.08 | 127.49 | Republic of Korea |  | KY296036 | KY295949 |  |  |  |  | Borzée et al. (2017) |
| *Bufo sachalinensis* cf. *sachalinensis* | Bufo gargarizans 45 | 36.08 | 127.49 | Republic of Korea |  | KY296037 | KY295948 |  |  |  |  | Borzée et al. (2017) |
| *Bufo sachalinensis* cf. *sachalinensis* | Bufo gargarizans 46 | 36.08 | 127.49 | Republic of Korea |  | KY296038 | KY295947 |  |  |  |  | Borzée et al. (2017) |
| *Bufo gargarizans* | JX5 | 28.7 | 115.8 | China: Jiangxi, Xinjian |  | DQ288692 |  |  |  |  |  | Hu et al. (2007) |
| *Bufo gargarizans* | FJAH | 27.7 | 118 | China: Fujian, Wuyishan |  | DQ288693 |  |  |  |  |  | Hu et al. (2007) |
| *Bufo gargarizans* | FJ11 | 27.7 | 118 | China: Fujian, Wuyishan |  | DQ288694 |  |  |  |  |  | Hu et al. (2007) |
| *Bufo gargarizans* | HB1 | 29.82 | 112.55 | China: Hubei, Shishou |  | DQ288696 |  |  |  |  |  | Hu et al. (2007) |
| *Bufo gargarizans* | JS11 | 33.38 | 120.13 | China: Jiangsu, Yancheng |  | DQ288697 |  |  |  |  |  | Hu et al. (2007) |
| *Bufo gargarizans* | SD2 | 36.18 | 118.17 | China: Shandong, Yiyuan |  | DQ288698 |  |  |  |  |  | Hu et al. (2007) |
| *Bufo gargarizans* | AH26 | 30.2 | 118.1 | China: Anhui, Huangshan |  | DQ288699 |  |  |  |  |  | Hu et al. (2007) |
| *Bufo gargarizans* | JS10 | 33.38 | 120.13 | China: Jiangsu, Yancheng |  | DQ288700 |  |  |  |  |  | Hu et al. (2007) |
| *Bufo gargarizans* | JSAH | 33.38 | 120.13 | China: Jiangsu, Yancheng |  | DQ288701 |  |  |  |  |  | Hu et al. (2007) |
| *Bufo gargarizans* | SD1 | 36.18 | 118.17 | China: Shandong, Yiyuan |  | DQ288702 |  |  |  |  |  | Hu et al. (2007) |
| *Bufo gargarizans* | SD3 | 36.18 | 118.17 | China: Shandong, Yiyuan |  | DQ288703 |  |  |  |  |  | Hu et al. (2007) |
| *Bufo gargarizans* | HN1 | 30.1 | 110.8 | China: Hunan, Shimen |  | DQ288704 |  |  |  |  |  | Hu et al. (2007) |
| *Bufo gargarizans* | YNAH | 24.27 | 102.73 | China: Yunnan, Jiangchuan |  | DQ288705 |  |  |  |  |  | Hu et al. (2007) |
| *Bufo gargarizans* | HN4 | 30.1 | 110.8 | China: Hunan, Shimen |  | DQ288706 |  |  |  |  |  | Hu et al. (2007) |
| *Bufo gargarizans* | JS7 | 33.38 | 120.13 | China: Jiangsu, Yancheng |  | DQ288707 |  |  |  |  |  | Hu et al. (2007) |
| *Bufo gargarizans* | HN8 | 30.1 | 110.8 | China: Hunan, Shimen |  | DQ288708 |  |  |  |  |  | Hu et al. (2007) |
| *Bufo gargarizans* | GZLN | 28.33 | 106.2 | China: Guizhou, Xishui |  | DQ288709 |  |  |  |  |  | Hu et al. (2007) |
| *Bufo gargarizans* | LN1 | 41.8 | 123.38 | China: Liaoning, Shenyang |  | DQ288710 |  |  |  |  |  | Hu et al. (2007) |
| *Bufo gargarizans* | GSBH | 36.03 | 103.73 | China: Gansu, Lanzhou |  | DQ288711 |  |  |  |  |  | Hu et al. (2007) |
| *Bufo gargarizans* | GS42 | 36.03 | 103.73 | China: Gansu, Lanzhou |  | DQ288712 |  |  |  |  |  | Hu et al. (2007) |
| *Bufo gargarizans* | GS45 | 36.03 | 103.73 | China: Gansu, Lanzhou |  | DQ288713 |  |  |  |  |  | Hu et al. (2007) |
| *Bufo gargarizans* | GS34 | 36.03 | 103.73 | China: Gansu, Lanzhou |  | DQ288714 |  |  |  |  |  | Hu et al. (2007) |
| *Bufo gargarizans* | SC2 | 29.6 | 103.4 | China: Sichuan, E'mei |  | DQ288715 |  |  |  |  |  | Hu et al. (2007) |
| *Bufo gargarizans* | LN6 | 41.8 | 123.38 | China: Liaoning, Shenyang |  | DQ288716 |  |  |  |  |  | Hu et al. (2007) |
| *Bufo gargarizans* | HLJHBGS | 45.75 | 126.63 | China: Heilongjiang, Harbin |  | DQ288717 |  |  |  |  |  | Hu et al. (2007) |
| *Bufo gargarizans* | HLJ3 | 45.75 | 126.63 | China: Heilongjiang, Harbin |  | DQ288718 |  |  |  |  |  | Hu et al. (2007) |
| *Bufo gargarizans* | GS41 | 36.03 | 103.73 | China: Gansu, Lanzhou |  | DQ288719 |  |  |  |  |  | Hu et al. (2007) |
| *Bufo gargarizans* | YN3 | 24.27 | 102.73 | China: Yunnan, Jiangchuan |  | DQ288720 |  |  |  |  |  | Hu et al. (2007) |
| *Bufo gargarizans* | CAS 242154 |  |  | China: Yunnan | KF665342 |  |  |  |  |  |  | Liedtke et al. (2016) |
| *Bufo gargarizans* | ZMMSU A-4127 |  |  | China: Yunnan | FJ882843 | FJ882843 | FJ882843 |  |  |  |  | Van Bocxlaer et al. (2010) |
| *Bufo gargarizans* | IEBR:A2015_62 | 23.02 | 104.51 | Vietnam: Thang Village, Tung Vai Commune, Quan Ba District, Ha Giang Province | LC155912 |  |  |  |  |  |  | Pham et al. (2016) |
| *Bufo gargarizans* | NIBRAM0000100276 |  |  | Gangwon-do Cheorwon-gun Gimhwa-eup | JQ815291 |  |  |  |  |  |  | Jeong et al. (2013) |
| *Bufo gargarizans* | NIBRAM0000100413 |  |  | Gyeongsangnam-do Goseong-gun Donghae- myeon | JQ815292 |  |  |  |  |  |  | Jeong et al. (2013) |
| *Bufo gargarizans miyakonis* |  | 24.77 | 125.325 | Japan: Okinawa, Miyako island |  |  |  |  |  |  |  | Igawa et al. (2006) |
| *Bufo tibetanus* | KIZ-97L004 | 27.87 | 100.148 | China: Yunnan, Zhongdian |  | AF190249 |  |  |  |  |  | Liu et al. (2000) |
| *Bufo tibetanus* | KIZ-97001 |  |  |  |  | AY924309 | AY936838 |  |  |  |  | Fu et al. (2005) |
| *Bufo andrewsi* |  |  |  |  |  |  | DQ158269 |  |  |  | DQ283905 | Frost et al. (2006) |
| *Bufo andrewsi* |  |  |  |  |  |  | DQ158270 | DQ158353 | DQ306531 |  |  | Pramuk et al. (2006) |
| *Bufo stejnegeri* |  |  |  | Republic of Korea |  | KR136211 | KR136211 |  |  |  |  | Dong and Yang (2016) |
| *Bufo japonicus* |  |  |  |  |  |  |  |  |  |  | AB612061 | Kurabayashi et al. (2011) |
| *Bufo japonicus formosus* |  | 40.34 | 140.34 | Japan: Aomori prefecture, Hirosaki city environs, Hirakawa | JN653294 |  |  | JN653307 |  |  | JN653314 | Recuero et al. (2012) |
| *Bufo japonicus formosus* |  | 35.43 | 139.45 | Japan: Tokyo prefecture, Tokyo city, Sendagi | JN653293 |  |  | JN653306 |  |  | JN653313 | Recuero et al. (2012) |
| *Bufo torrenticola* |  | 34.159 | 136.037 | Japan: Nara, Odaigahara |  |  |  |  |  |  |  | Igawa et al. (2006) |
| *Bufo torrenticola* | BTorrBB100 | 34.14 | 135.51 | Japan: Nara prefecture, Tenkawa environs | JN653292 |  |  | JN653305 |  |  | JN653312 | Recuero et al. (2012) |
| *Bufo tuberculatus* |  | 27.823 | 99.72 | China: Yunnan, Zhongdian |  |  |  |  |  |  |  | Liu et al. (2000) |
| *Bufo “Torrentophryne” pageoti* | CAS233251 | 22.972 | 93.645 | Myanmar: Chin State, Falam Township, Laiva village | KF665335 |  |  |  | KF666231 |  | KF665978 | Liedtke et al. (2016) |
| *Bufo “Torrentophryne” pageoti* | CAS233299 |  |  | Myanmar: Chin State |  |  | KU183330 | KU183135 |  |  |  | Wogan et al. (2016) |
| *Bufo “Torrentophryne” pageoti* | CAS233176 |  |  | Myanmar: Chin State |  |  | KU183329 | KU183140 |  |  |  | Wogan et al. (2016) |
| *Bufo “Torrentophryne”*  *tuberospinia* | KIZ91A089 |  |  |  |  | AF190255 |  |  |  |  |  | Liu et al. (2000) |
| *Bufo “Torrentophryne”*  *aspinia* | KIZ93A011 |  |  |  | AF160787 | AF190254 |  |  |  |  |  | Liu et al. (2000) |
| *Bufo “Torrentophryne”*  *cryptotympanicus* | AMNH 13198 |  |  |  | AF160789 |  |  |  |  |  |  | Liu et al. (2000) |
| *Epidalea calamita* | Isolate 169 |  |  |  | DQ629607 | DQ629617 |  |  |  |  |  | Stöck et al. (2006) |
| *Epidalea calamita* |  | 36.333 | 5.817 | Spain: Cadiz Prov., Andalusia, 3.1 km S Benalup de Sidonia on road to Vejer de La Frontera |  |  |  |  |  |  |  | Stöck et al. (2006) |
| *Strauchbufo raddei* |  | 35.95 | 103.27 | Liujiaxia, Gansu Province, China | KT223827 | KT223827 | KT223827 |  |  |  |  | Zhang et al. (2016) |
| *Bufo eichwaldi* |  | 38.33 | 48.47 | Azerbaijan: Astara district, Kizhaba environs, Talysh mts | JN647239 |  |  | JN646955 |  |  | JN646906 | Recuero et al. (2012) |
| *Bufo eichwaldi* |  | 38.39 | 48.38 | Azerbaijan; Lerik district, Agoshapeshta env., Talysh mts | JN647240 |  |  | JN646953 |  |  | JN646908 | Recuero et al. (2012) |
| *Bufo bufo* | Isolate B2 | 45.621 | 13.869 | Italy: Trieste, San Dorligo della Valle | EU497414 | EU497515 |  |  |  |  |  | Stöck et al. (2008) |
| *Bufo bufo* | Isolate B1 | 44.76 | 14.42 | Croatia: Cres Island, Belej | EU497413 | EU497513 |  |  |  |  |  | Stöck et al. (2008) |
| *Bufo bufo* |  | 49.54 | 3.5 | France: Erloy, Foret de Regnaval | JN647129 |  |  | JN646935 |  |  | JN646884 | Recuero et al. (2012) |
| *Bufo bufo* |  | 64.32 | 40.41 | Russia: Arkhangelsk province, Arkhangelsk environs | JN647113 |  |  | JN646941 |  |  | JN646893 | Recuero et al. (2012) |
| *Bufo bufo* |  | 52.18 | 5.49 | Netherlands: Elspeet | JN647012 |  |  | JN646916 |  |  | JN646866 | Recuero et al. (2012) |
| *Bufo verrucosissimus* |  | 43.55 | 40.39 | Russia: Krasnodar territory, Caucasian Nature Reserve, environs ofChernorechye | JN647235 |  |  | JN646961 |  |  | JN646915 | Recuero et al. (2012) |
| *Bufo spinosus* | BspinBB119 | 33.31 | 5.6019 | Morocco: Ifrane | JN647215 |  |  | JN646946 |  |  | JN646899 J | Recuero et al. (2012) |
| *Bufo eichwaldi* | Isolate 130 |  |  |  | JQ348826 |  | JQ348546 |  |  |  |  | Garcia-Porta et al. (2012) |
| *Bufo eichwaldi* | Isolate 131 |  |  |  | JQ348825 |  | JQ348545 |  |  |  |  | Garcia-porta et al. (2012) |
| *Bufo eichwaldi* |  |  |  | Avrora (Azerbaijan) |  |  | JQ348546 |  |  |  |  | Garcia-porta et al. (2012) |
| *Bufo spinosus* | 1 | 42.42 | -3.71 | Spain |  |  | JQ348501 |  |  |  |  | Garcia-porta et al. (2012) |
| *Bufo spinosus* | 2 | 43.12 | -3.71 | Spain |  |  | JQ348497 |  |  |  |  | Garcia-porta et al. (2012) |
| *Bufo spinosus* | 3 | 43.12 | -3.72 | Spain |  |  | JQ348527 |  |  |  |  | Garcia-porta et al. (2012) |
| *Bufo spinosus* | 6 | 42.34 | 1.76 | Spain |  |  | JQ348529 |  |  |  |  | Garcia-porta et al. (2012) |
| *Bufo spinosus* | 9 | 41.92 | 0.72 | Spain |  |  | JQ348486 |  |  |  |  | Garcia-porta et al. (2012) |
| *Bufo spinosus* | 10 | 42.37 | 2.98 | Spain |  |  | JQ348485 |  |  |  |  | Garcia-porta et al. (2012) |
| *Bufo spinosus* | 12 | 38.79 | -9.39 | Portugal |  |  | JQ348506 |  |  |  |  | Garcia-porta et al., (2012) |
| *Bufo spinosus* | 19 | 43.86 | 3.38 | France |  |  | JQ348481 |  |  |  |  | Garcia-porta et al. (2012) |
| *Bufo spinosus* | 42 | 40.28 | -5.25 | Spain |  |  | JQ348512 |  |  |  |  | Garcia-porta et al. (2012) |
| *Bufo spinosus* | 43 | 40.28 | -5.25 | Spain |  |  | JQ348513 |  |  |  |  | Garcia-porta et al. (2012) |
| *Bufo spinosus* | 44 | 40.28 | -5.25 | Spain |  |  | JQ348511 |  |  |  |  | Garcia-porta et al. (2012) |
| *Bufo bufo* | 64 | 44.11 | 15.23 | Croatia |  |  | JQ348456 |  |  |  |  | Garcia-porta et al. (2012) |
| *Bufo bufo* | 66 | 40.37 | 15.53 | Italy |  |  | JQ348466 |  |  |  |  | Garcia-porta et al. (2012) |
| *Bufo bufo* | 68 | 52.7 | 1.39 | United Kingdom |  |  | JQ348445 |  |  |  |  | Garcia-porta et al. (2012) |
| *Bufo bufo* | 69 | 39.61 | 19.78 | Greece |  |  | JQ348432 |  |  |  |  | Garcia-porta et al. (2012) |
| *Bufo bufo* | 83 | 50.76 | 4.28 | Belgium |  |  | JQ348470 |  |  |  |  | Garcia-porta et al. (2012) |
| *Bufo verrucosissimus* | 121 | 41.65 | 41.8 | Russia |  |  | JQ348416 |  |  |  |  | Garcia-porta et al. (2012) |
| *Bufo verrucosissimus* | 123 | 41.82 | 46.27 | Georgia |  |  | JQ348417 |  |  |  |  | Garcia-porta et al. (2012) |
| *Bufo verrucosissimus* | Osmaniye 1 |  |  | Turkey |  |  |  |  | MN433045 |  |  | Özdemir et al. (2020) |
| *Bufo eichwaldi* | 130 | 38.45 | 48.73 | Azerbaijan |  |  | JQ348546 |  |  |  |  | Garcia-porta et al. (2012) |
| *Bufo eichwaldi* | 131 | 38.65 | 48.8 | Azerbaijan |  |  | JQ348545 |  |  |  |  | Garcia-porta et al. (2012) |
| *Bufo spinosus* | 132 | 35.37 | -5.54 | Morocco |  |  | JQ348541 |  |  |  |  | Garcia-porta et al. (2012) |
| *Bufo spinosus* | 141 | 34.95 | -5.23 | Morocco |  |  | JQ348533 |  |  |  |  | Garcia-porta et al. (2012) |
| *Bufo spinosus* | 144 | 36.73 | 8.71 | Tunisia |  |  | JQ348532 |  |  |  |  | Garcia-porta et al. (2012) |
| *Bufotes boulengeri* | MVZ235680 (166) | 33.92 | 3.13 | Tunisia: Nefta oasis, Tawzar |  | DQ629721 | DQ629602 |  |  |  |  | Stöck et al. (2006) |
| *Bufotes viridis* | MVZ164718 | 47.85 | 16.83 | Austria: 3.2 km E Podersdorf Buraenland |  | DQ629686 | DQ629606 |  |  |  |  | Stöck et al. (2006) |
| *Bufotes sitibundus* | MVZ230208-238 | 40.17 | 29.08 | Turkey: Bursa Province |  | DQ629623 | DQ629600 |  |  |  |  | Stöck et al. (2006) |
| *Bufotes luristanicus* | MTKDD43943-58 | 29.2 | 53.33 | Iran: Posht Chenar |  | DQ629614 | DQ629610 |  |  |  |  | Stöck et al. (2006) |
| *Bufotes viridis* | NME 974/02-90/91 | 51.42 | 12.88 | Germany: Thuringia |  | DQ629672 | DQ629673 |  |  |  |  | Stöck et al. (2006) |
| *Bufotes latastii* | 81 | 35.28 | 75.62 | Pakistan: Satpara Lake |  | DQ629848 | DQ629599 |  |  |  |  | Stöck et al. (2006) |
| *Bufotes oblongus* |  |  |  |  | KT031477 |  |  |  | KT031667 |  |  | Portik and Papenfuss (2012) |
| *Bufotes oblongus* |  |  |  |  | KT031476 |  |  | DQ158270 | DQ158353 |  |  | Portik and Papenfuss (2012) |
| *Bufotes balearicus* |  | 40.723 | 8.342 | Italy: Sardinia, Sassari, Monte Nurra |  |  |  |  |  |  |  | Stöck et al. (2008) |
| *Bufotes perrini* | MS-2006 isolate 304 | 35.967 | 56.068 | Iran: Delbar Field Station, Touran Protected Area |  | DQ629792 | DQ629605 |  |  |  |  | Stöck et al. (2008) |
| *Bufotes viridis* | isolate 168 | 47.85 | 16.833 | Austria: Podersdorf Burgenland |  | DQ629686 | DQ629606 |  |  |  |  | Stöck et al. (2006) |
| *Bufotes viridis* | AMNH A168402 |  |  |  |  |  |  |  |  | DQ283940 |  | Frost et al. 2006 |
| *Bufotes sitibundus* | 1813PsVi |  |  |  | FJ882812 | FJ882812 | FJ882812 |  |  |  | FJ882713 | Van Bocxlaer et al. (2010) |
| *Bufotes sitibundus* | MVZ 230208 | 40.167 | 29.083 | Turkey: Osman Gazi, Bursa, Bursa Prov |  | DQ629623 | DQ629600 |  |  |  |  | Stöck et al. (2006) |
| *Bufotes luristanicus* | MTKD D 43943 | 29.2 | 53.333 | Iran: Posht Chenar |  | DQ629614 | DQ629610 |  |  |  |  | Stöck et al. (2006) |
| *Bufotes luristanicus* | NP B-13-1 |  |  |  | GU226835 | GU226835 | GU226835 |  |  |  | GU226833 | Van Bocxlaer et al. (2010) |
| *Bufotes latastii* | ZMB 62723 | 35.283 | 75.617 | Pakistan: Northern Areas (Baltistan), Himalaya, Satpara river |  | DQ629848 | DQ629599 |  |  |  |  | Stöck et al. (2006) |
| *Bufotes pewzowi* | CAS 197008 | 37.78 | 75.23 | China: Northwestern, Taxkurgan, E-Pamir |  | DQ629758 | DQ629603 |  |  |  |  | Stöck et al. (2006) |
| *Anaxyrus cognatus* | MVZ 143048 |  |  |  | AY680230 |  |  |  |  |  |  | Pauly et al. (2004) |
| *Anaxyrus cognatus* | BF181 |  |  |  |  |  | HM135379 |  |  |  |  | Fontenot et al. (2011) |
| *Anaxyrus cognatus* | BF182 |  |  |  |  |  | HM135380 |  |  |  |  | Fontenot et al. (2011) |
| *Anaxyrus cognatus* | BF183 |  |  |  |  |  | HM135381 |  |  |  |  | Fontenot et al. (2011) |
| *Anaxyrus terrestris* | AMNH A168433 |  |  |  | DQ283158 |  |  |  |  |  |  | Frost et al. (2006) |
| *Anaxyrus hemiophrys* | MVZ 137738 |  |  |  | AY680213 |  |  |  | KJ609652 | FJ004269 |  | Brandvain et al. (2014) |
| *Anaxyrus boreas* | GBP 556 |  |  |  |  | EU938415 |  |  |  |  |  | Pauly et al. 2006 (unpublished) |
| *Rhinella abei* | CFBH18141 |  |  | Brazil: Quatro Barras |  |  |  |  |  | GU907382 |  | Thomé et al. (2010) |
| *Rhinella marinus* |  |  |  | Southern America |  |  |  |  |  | U59922.1 |  | Fyhrquist et al. (1998) |
| *Rhinella pombali* | MNRJ38327 |  |  | Catas Altas, Minas Gerais |  | GU907179 | GU907317 |  |  | GU907411 |  | Thomé et al. (2010) |
| *Rhinella pombali* | CFBH14647 |  |  | Cristina, Minas Gerais | GU907220 | GU907151 | GU907289 |  |  | GU907366 |  | Thomé et al. (2010) |
| *Rhinella pombali* | CFBH9937 |  |  | Cristina, Minas Gerais | GU907198 | GU907129 | GU907267 |  |  | GU907338 |  | Thomé et al. (2010) |
| *Rhinella pombali* | MTR11543 |  |  | Brazil: Santa Marta, Minas Gerais |  |  |  |  |  | GU907392 |  | Thomé et al. (2010) |
| *Rhinella ornatus* | CFBH18808 |  |  | Brazil: Teresópolis, Rio de Janeiro | GU907255 | GU907186 | GU907324 |  |  |  |  | Thomé et al. (2010) |
| *Rhinella ornatus* | CFBH7193 |  |  | Brazil: S. Antônio do Pinhal, São Paulo | GU907192 | GU907123 | GU907261 |  |  | GU907332 |  | Thomé et al. (2010) |
| *Rhinella ornatus* | CFBH13792 |  |  | Brazil: Peruíbe, São Paulo | GU907223 | GU907154 | GU907292 |  |  | GU907368 |  | Thomé et al. (2010) |
| *Rhinella ornatus* | CFBH2865 |  |  | Brazil: Aracruz, Espírito Santo | GU907207 | GU907138 | GU907276 |  |  | GU907344 |  | Thomé et al. (2010) |
| *Rhinella fernandezae* | LGE 8716 |  |  |  | KP685204 |  |  |  |  |  |  | Pereyra et al. (2016) |
| *Rhinella schneideri* | VUB 1965 |  |  |  | FJ882831 |  |  |  |  |  |  | Pauly et al. (2004) |
| *Incilius luetkenii* | UTA:A-50877 |  |  |  |  |  | JN868025 |  |  |  |  | Mendelson et al. (2011) |
| *Incilius occidentalis* | UTA:A-13543 |  |  |  |  |  | JN868033 |  |  |  |  | Mendelson et al. (2011) |
| *Incilius pisinnus* | UTA:A-JAC 26118 |  |  |  |  |  | JN868004 |  |  |  |  | Mendelson et al. (2011) |
| *Incilius valliceps* | MZFC JRM-3868 |  |  |  |  |  | JN868041 |  |  |  |  | Mendelson et al. (2011) |
| *Incilius valliceps* |  |  |  |  |  | HM563855 |  |  |  |  |  | Mendelson et al. (2011) |
| *Incilius nebulifer* | TNHC 62000 |  |  | USA: Texas, San Saba County, Colorado Bend State Park |  |  | HQ290945 |  |  |  |  | Santos and Canatella (2012) |
| *Incilius nebulifer* | NCx3i1 |  |  |  |  |  |  |  |  | EF372223 |  | Vogel and Johnson (2008) |
| *Incilius bocourti* |  |  |  |  | AY680246 |  |  |  |  |  |  | Pauly et al. (2004) |
| *Incilius bocourti* | UTA A-50920 |  |  |  |  |  |  | JN868008 |  |  |  | Mendelson et al. (2011) |
| *Incilius bocourti* |  |  |  |  | AY680246 |  |  |  | KJ609662 |  |  | Brandvain et al. (2014) |
| *Incilius ibarrai* |  |  |  |  | AY680249 |  |  |  |  |  |  | Pauly et al. (2004) |
| *Incilius mazatlanensis* |  |  |  |  | AY680254 |  |  |  |  |  |  | Pauly et al. (2004) |
| *Duttaphrynus parietalis* |  |  |  |  | FJ882784 |  |  |  |  |  |  | Van Bocxlaer et al. (2009) |
| *Duttaphrynus stomaticus* |  |  |  |  | KJ532264 |  |  |  |  |  |  | Brandvain et al. (2014) |
| *Duttaphrynus brevirostris* | SDB 4714 |  |  |  | FJ882786 |  |  |  |  |  |  | Van Bocxlaer et al. (2009) |
| *Duttaphrynus crocus* | CAS 220193 |  |  |  | FJ882789 |  |  |  |  |  |  | Van Bocxlaer et al. (2009) |
| *Duttaphrynus hololius* | SDB 4240 |  |  |  | FJ882781 |  |  |  |  |  |  | Van Bocxlaer et al. (2009) |
| *Duttaphrynus stuarti* | CAS 221485 |  |  |  | FJ882788 |  |  |  |  |  |  | Van Bocxlaer et al. (2009) |
| *Ingerophrynus galeatus* | ROM 33281 |  |  |  | AF160781 |  |  |  |  |  |  | Liu et al. (2000) |

**Supplementary file 1K.**

**Information of primers used to amplify targeted gene fragments.** Some specific primers designed in this study using Primer3 v.0.4.0 (Untergasser et al. 2012).

| **Gene fragment** | **Primer** | **Sequence (5' to 3')** | **Source** | **Annealing temperature (°C)** |
| --- | --- | --- | --- | --- |
| Control region | Control B-H | GTCCATTGGAGGTTAAGATCTACCA | Goebel et al. (1999) | 55 |
| Control region | CR-BGarF | TTGGACGATAGCAAGGAACACTC | Borzée et al. (2017) | 55 |
| Control region | CR-BGarR | CCTGACTTCTCTGAGGCCGCTTT | Borzée et al. (2017) | 55 |
| Control region | conBG-L | GCACGATAGCAAGGAACAC | Liu et al. (2000) | 45 |
| Control region | conBG-H | CCGCTTTAAGGTACGATA | Liu et al. (2000) | 45 |
| *ND1-ND2* | ND1-L-int | CGAGCATCC TACCCACGATTTCG | Fu et al. (2005) | 50 |
| *ND1-ND2* | ND1-H4980 | ATT TTTCGTAGTTGGGTTTGRTT | Macey et al. (1998) | 50 |
| *ND2* | BGND2F | TCTCATTCCCAATCTCACTTCTACT | This study | 50 |
| *ND2* | BGND2R | GCC TCACCCTCCGACAATA | This study | 50 |
| *POMC* | POMC DRV F1 | ATATGTCATGASCCAYTTYCGCTGGAA | Vieites et al. (2009) | 58 |
| *POMC* | POMC DRV R1 | GGCRTTYTTGAAWAGAGTCATTAGWGG | Vieites et al. (2009) | 58 |
| *RAG-1* | snoBGRAG1F | TGAGAAACGCAGAGAAAGCCC | This study | 60 |
| *RAG-2* | snoBGRAG1R | GACGGGTGGCATCACAAAGAG | This study | 60 |
| *Rho* | BGRho01-F | CGACTACACCCTGAAGCC | This study | 50 |
| *Rho* | BGRho01-R | CCAACAGATAAGGAAGAAGACCAC | This study | 50 |

**Supplementary file 1L.**

**Most probable unlinked site models for species tree reconstruction on each multi-locus.** The model was selected with jModelTest 2 v.2.1.10 (Darriba et al. 2015).

| **Tree** | **Locus** | **Specimens number and sequences length (bp)** | **Selected unlinked model site** |
| --- | --- | --- | --- |
| Species tree  (unlinked multi-loci) | *CR* | 90 (369 bp) | HKY+G |
|  | *ND2* | 80 (383 bp) | GTR +G |
|  | *RAG-1* | 39 (309 bp) | GTR +G |
|  | *16S* | 29 (544 bp) | GTR +G |
|  | *CXCR4* | 11 (570 bp) | JC |
|  | *POMC* | 37 (284 bp) | GTR |
|  | *Rho* | 27 (207 bp) | GTR |


**Supplementary file 1M.**

**Best substitution models for sequences evolution of each mtDNA and nuclear data.** The partition recovered with Partition Finder v.2.1.1 (Lanfear et al. 2017).

| **Tree**  **(BI and ML)** | **Locus** | **Specimens number (*n* taxa) and sequences length (bp)** | **Partition strategy based on type of coding sequence** | **Evolutionary model** |
| --- | --- | --- | --- | --- |
| *16S* gene | *16S* | 78 (407 bp) | Non-coding: 1-407 bp | GTR+I+G |
| Concatenated mtDNA  (894 bp) | *CR* | 221 (357 bp) | Non-coding: 1-357 bp | HKY+G |
|  | *ND2* | 221 (537 bp) | Partial 5’ intron 1: 358-572 bp | HKY+I |
|  |  |  | Exon 1 by codon position: 573/574/575-824 bp | HKY, GTR+G and K80+I |
|  |  |  | Partial 3’ intron 2: 825-894 bp | GTR+G |
| Concatenated nuclear (1030 bp) | *POMC* | 44 (354 bp) | Partial 5’ intron:  1-76 bp | K80+I |
|  |  |  | Partial 3’ Exon by codon position:  77/78/79-354 bp | K80, F81 and F81+I |
|  | *RAG-1* | 44 (677 bp) | Partial exon by codon position:  355/356/357-805 bp | JC+I, F81 and F81 |
|  | *Rho* | 44 (225 bp) | Partial 5’ intron:  806-919 bp | K80+I |
|  |  |  | Exon by codon position:  920/921/922-1030 bp | JC+I, K80+I+G and JC |

**Supplementary file 1N.**

Calculated likelihood values for Homogeneity test analysed on ML tree built from mtDNA and nuclear data.

| **Dataset (locus)** | **Likelihood trees**  **(degree of freedom)** | **Homogeneity test** | | |
| --- | --- | --- | --- | --- |
| mtDNA *CR-ND2* | 130 | -Ln_A_ | Ti/Tv | kappa |
|  |  | 18893.867 | 2.739 | 5.818 |
|  |  | –Ln_0_ | Ti/Tv | kappa |
|  |  | 20301.805 | 2.701 | 5.739 |
| nuDNA *POMC-RAG-1-Rho* |  | -Ln_A_ | Ti/Tv | kappa |
|  | 40 | 2504.100 |  |  |
|  |  | –Ln_0_ | Ti/Tv | kappa |
|  |  | 2472.814 |  |  |

**Supplementary file 1O.**

The datasets used in the reconstruction of paleogeographic maps in this study.

| **Dataset** | **Reference** |
| --- | --- |
| Diva-GIS Elevation data by country in East Asia | (Hijmans et al., 2005) |
| WorldClim v. 1.4 datasets of 19 bioclimatic variables derived from current temperature and precipitation:  BIO1 = Annual Mean Temperature  BIO2 = Mean Diurnal Range (Mean of monthly (max temp - min temp))  BIO3 = Isothermality (BIO2/BIO7) (×100)  BIO4 = Temperature Seasonality (standard deviation ×100)  BIO5 = Max Temperature of Warmest Month  BIO6 = Min Temperature of Coldest Month  BIO7 = Temperature Annual Range (BIO5-BIO6)  BIO8 = Mean Temperature of Wettest Quarter  BIO9 = Mean Temperature of Driest Quarter  BIO10 = Mean Temperature of Warmest Quarter  BIO11 = Mean Temperature of Coldest Quarter  BIO12 = Annual Precipitation  BIO13 = Precipitation of Wettest Month  BIO14 = Precipitation of Driest Month  BIO15 = Precipitation Seasonality (Coefficient of Variation)  BIO16 = Precipitation of Wettest Quarter  BIO17 = Precipitation of Driest Quarter  BIO18 = Precipitation of Warmest Quarter  BIO19 = Precipitation of Coldest Quarter | (Hijmans et al., 2005) |
| LGM shelves in Eurasia | (Ray et al., 2001) |
| >50m Glaciated area on Asian mountainous in LGM | (Kuhle, 2004) |
| Miocene tectonic plates models | (Matthews et al., 2016) |
| Tibetan-Himalayan mapping | (Styron et al., 2010; Taylor and Yin, 2009) |
| The Yellow Sea paleoclimate | IHO Sea Area (http://www.marineregions.org/ https://doi.org/10.14284/323) |
| The Yellow sea level depth during LGM | (Li et al., 2016) |
