# Supplementary material for: From Gondwana to the Yellow Sea, evolutionary diversifications of true toads Bufo sp. in the Eastern Palearctic and a revisit of species boundaries for Asian lineages
Source: eLife. 2022 Jan 28;11:e70494. doi: 10.7554/eLife.70494 (PMC8920510; doi:10.7554/eLife.70494)
Supplement: Supplementary file 2. [file elife-70494-supp2.docx]

**References and notes**

The dataset references for all citations in Supplementary file 1A and Supplementary file 1J.

| **Supplementary file 1A (Dataset references for authority, type localities of described species members of East Asian *Bufo* spp.)** | |
| --- | --- |
| **Citation** | **Bibliography** |
| Frost (2021) | D. R. Frost, Amphibian Species of the World: an Online Reference. Version 6.1 (9 March 2021). Electronic Database accessible at https://amphibiansoftheworld.amnh.org/index.php. *American Museum of Natural History, New York, USA* (2021), doi:doi.org/10.5531/db.vz.0001. |
| Rosenhof et al. (1758) | R. von Rosenhof, A. Johann, A. von Haller, *Historia Naturalis Ranarum Nostratium In Qua Omnes Earum Proprietates Præsertim Quæ Ad Generationem Ipsarum Pertinent, Fusius Enarrantur* (Nurnberg, 1758; http://lib.ugent.be/catalog/rug01:002032974). |
| Laurenti (1768) | J. N. Laurenti, *Specimen medicum, exhibens synopsin reptilium emendatum cum experimentis circa venena et antidota reptilium austriacorum* (Typ. Joan. Thom. Nob. de Trattnern, Wien, Austria, 1768). |
| Cantor (1842) | T. Cantor, General features of Chusan, with remarks on the flora and fauna of that island. *Annals and Magazine of Natural History*. **Series 1**, 481–493 (1842). |
| Matsui (1986) | M. Matsui, Geographic variation in Toads of the *Bufo bufo* complex from the Far East, with a description of a new subspecies. *Copeia*, 561–579 (1986). |
| Steindachner (1867) | F. Steindachner, in *1. Amphibien* (Resurrected by Channing, A. Howell, K.M. (2006) Amphibians of East Africa. Ithaca, New York: Cornell University Press, Wien: K. K. Hof- und Staatsdruckerei, 1867). |
| Nikolsky (1905) | A. M. Nikolsky, Presmykaiushchiiasia i zemnovodnyia rossiiskoi imperii [= Herpetologia rossica]. *Mémoires de l’Académie Impériale des Sciences de St. Pétersbourg*. **Série 8**, 1–518 (1905). |
| Milto and Barabanov (2011) | K. D. Milto, A. V. Barabanov, An annotated catalogue of the amphibian types in the collection of the Zoological Institute, Russian Academy of Sciences, St. Petersburg. *Russian Journal of Herpetology*. **18**, 137–153 (2011). |
| Schmidt (1925) | K. P. Schmidt, New Chinese amphibians and reptiles. *American Museum Novitates*. **175**, 1–3 (1925). |
| Zarevskij (1926) | S. F. Zarevskij, Notes on some Batracians from the Palaearctic region. *Annuaire du Musée Zoologique de l’Académie des Sciences de Leningrad*. **26**, 74–78 (1926). |
| Borkin and Matsui (1986) | L. J. Borkin, M. Matsui, in *Sistematika i ekologiia amfibii i reptilii*, N. B. Ananjeva, L. J. Borkin, Eds. (Zoologicheskii Instituta SSSR, Leningrad, 1986), pp. 43–53. |
| Barbour (1908) | T. Barbour, Some new reptiles and amphibians. *Bulletin of the Museum of Comparative Zoology*. **51**, 315–325 (1908). |
| Stejneger (1926) | L. Stejneger, A new toad from China. *J. Washington Acad. Sci.* **16**, 445–446 (1926). |
| Okada (1931) | Y. Okada, *The tailless batrachians of Japanese empire* (Imp. Agricult. Exp. Station, Nishighara, Tokyo, 1931). |
| Boulenger (1883) | G. A. Boulenger, Description of a new species of *Bufo* from Japan. *Proceedings of the Zoological Society of London*, 139–140 (1883). |
| Boie (1826) | H. Boie, Merkmale einiger japonischen Lurche. *Isis von Oken*. **18**, 203–215 (1826). |
| Temminck and Schlegel (1838) | C. J. Temminck, H. Schlegel, *Fauna Japonica sive Descriptio animalium, quae in itinere per Japonianum, jussu et auspiciis superiorum, qui summum in India Batava Imperium tenent, suscepto, annis 1823–1830 colleget, notis observationibus et adumbrationibus illustratis* (Leiden: J. G. Lalau, Volume 3 (., 1838). |
| Matsui (1986) | M. Matsui, A new toad from Japan. *Contributions From The Biological Laboratory, Kyoto University*. **25**, 1–10 (1976). |
| Liu and Hu (1962) | C. C. Liu, S. Q. Hu, A herpetological report of Kwangsi. *Acta Zoologica Sinica/ Dong wu xue bao*. **Beijing 14**, 73–104 (1962). |
| Rao and Yang (1994) | D. Q. Rao, D. T. Yang, The study of early development and evolution of *Torrentophryne aspinia*. *Zoological Research/Dōngwùxué yánjiū. Kunming*. **14**, 142–157 (1994). |
| Yang et al. (1996) | D.-T. Yang, W.-Z. Liu, D.-Q. Rao, A new toad genus of Bufonidae -*Torrentophryne* from Transhimalaya Mountain of Yunnan of China with Its biology. *Zoological Research/Dōngwùxué yánjiū*. **17**, 353–359 (1996). |
| Schmidt (1931) | K. P. Schmidt, A new toad from Korea. *Copeia*, 93–94 (1931). |
| Bourret (1937) | R. Bourret, Notes herpétologiques sur l’Indochine française. XIV. Les Batraciens de la collection du Laboratoire des Sciences Naturelles de l’Université. Descriptions de quinze espèces ou variétés nouvelles. *Annexe au Bulletin général de l’Instruction publique*. **4**, 5–56 (1937). |
| Stejneger (1907) | L. Stejneger, *Herpetology of Japan and adjacent territory* (Washington, 1907), vol. 58. |
| Okada (1966) | Y. Okada, *Fauna Japonica. Anura (Amphibia)* (Tokyo, 1966). |

| **Supplementary file 1J (Dataset references for East Asian *Bufo* spp. Genbank sequences)** | |
| --- | --- |
| **Citation** | **Bibiliography** |
| Yang et al. (2015) | J. Yang, J. Liu, R. Xue, L. Chen, Characterization of the mitochondrial genome of *Bufo gargarizans minshanicus* (Anura: Bufonidae). *Mitochondrial DNA*. **27**, 3327–3328 (2015). |
| Fu et al. (2005) | J. Fu, C. J. Weadick, X. Zeng, Y. Wang, Z. Liu, Y. Zheng, C. Li, Y. Hu, Phylogeographic analysis of the *Bufo gargarizans* species complex: A revisit. *Molecular Phylogenetics and Evolution*. **37**, 202–213 (2005). |
| Macey et al. (1998) | J. R. Macey, J. A. Schulte, A. Larson, Z. Fang, Y. Wang, B. S. Tuniyev, T. J. Papenfuss, Phylogenetic relationships of toads in the *Bufo bufo* species group from the eastern escarpment of the Tibetan Plateau: a case of vicariance and dispersal. *Molecular Phylogenetics and Evolution*. **9**, 80–87 (1998). |
| Borzée et al. (2017) | A. Borzée, J. L. Santos, S. Sánchez-RamÍrez, Y. Bae, K. Heo, Y. Jang, M. J. Jowers, Phylogeographic and population insights of the Asian common toad (*Bufo gargarizans*) in Korea and China: population isolation and expansions as response to the ice ages. *PeerJ*. **5**, e4044 (2017). |
| Hu et al. (2007) | Y. L. Hu, X. B. Wu, Z. G. Jiang, P. Yan, X. Su, S. Y. Cao, Population genetics and phylogeography of *Bufo gargarizans* in China. *Biochemical Genetics*. **45**, 697–711 (2007). |
| Liedtke et al. (2016) | H. C. Liedtke, H. Müller, M. O. Rödel, M. Menegon, L. G. N. Gonwouo, M. F. Barej, V. Gvoždík, A. Schmitz, A. Channing, P. Nagel, S. P. Loader, No ecological opportunity signal on a continental scale? Diversification and life-history evolution of African true toads (Anura: Bufonidae). *Evolution*. **70**, 1717–1733 (2016). |
| Van Bocxlaer et al. (2010) | I. Van Bocxlaer, S. P. Loader, K. Roelants, S. D. Biju, M. Menegon, F. Bossuyt, Gradual adaptation toward a range-expansion phenotype initiated the global radiation of toads. *Science*. **327**, 679–682 (2010). |
| Pham et al. (2015) | C. T. Pham, T. Q. Nguyen, M. Bernardes, T. T. Nguyen, T. Ziegler, First records of Bufo gargarizans Cantor, 1842 and *Odorrana lipuensis* Mo, Chen, Wu, Zhang et Zhou, 2015 (Anura: Bufonidae, ranidae) from Vietnam. *Russian Journal of Herpetology*. **23**, 103–107 (2016). |
| Jeong et al. (2013) | T. J. Jeong, J. Jun, S. Han, H. T. Kim, K. Oh, M. Kwak, DNA barcode reference data for the Korean herpetofauna and their applications. *Molecular Ecology Resources*. **13**, 1019–1032 (2013). |
| Igawa et al. (2006) | T. Igawa, A. Kurabayashi, M. Nishioka, M. Sumida, Molecular phylogenetic relationship of toads distributed in the Far East and Europe inferred from the nucleotide sequences of mitochondrial DNA genes. *Molecular Phylogenetics and Evolution*. **38**, 250–260 (2006). |
| Liu et al. (2000) | W. Liu, A. Lathrop, J. Fu, D. Yang, R. W. Murphy, Phylogeny of East Asian bufonids inferred from mitochondrial DNA sequences (Anura: Amphibia). *Molecular Phylogenetics and Evolution*. **14**, 423–435 (2000). |
| Frost et al. (2006) | D. Frost, T. Grant, J. Faivovich, A. Bain Raoul, Haas, C. Haddad, R. De Sá, A. Channing, M. Wilkinsin, S. Donnellan, C. Raxworthy, J. Campbell, B. Blotto, P. Moler, R. Drewes, R. Nussbaun, J. Lynch, D. Green, W. Wheeler, The Amphibian Tree of Life. *Bulletin of the American Museum of Natural History*. **297**, 1–370 (2006). |
| Pramuk et al. (2006) | J. B. Pramuk, Phylogeny of South American *Bufo* (Anura: Bufonidae) inferred from combined evidence. *Zoological Journal of the Linnean Society*. **146**, 407–452 (2006). |
| Dong and Yang (2016) | B. Dong, B. Yang, The complete mitochondrial genome of the *Bufo stejnegeri* (Anura: Bufonidae). *Mitochondrial DNA*. **27**, 2885–2886 (2016). |
| Kurabayashi et al. (2011) | A. Kurabayashi, M. Matsui, D. M. Belabut, H. Sen Yong, N. Ahmad, A. Sudin, M. Kuramoto, A. Hamidy, M. Sumida, From Antarctica or Asia? New colonization scenario for Australian-New Guinean narrow mouth toads suggested from the findings on a mysterious genus *Gastrophrynoides*. *BMC Evolutionary Biology*. **11**, 175 (2011). |
| Recuero et al. (2012) | E. Recuero, D. Canestrelli, J. Vörös, K. Szabó, N. A. Poyarkov, J. W. Arntzen, J. Crnobrnja-Isailovic, A. A. Kidov, D. Cogâlniceanu, F. P. Caputo, G. Nascetti, I. Martínez-Solano, Multilocus species tree analyses resolve the radiation of the widespread *Bufo bufo* species group (Anura, Bufonidae). *Molecular Phylogenetics and Evolution*. **62**, 71–86 (2012). |
| Wogan et al. (2016) | G. O. U. Wogan, B. L. Stuart, D. T. Iskandar, J. A. McGuire, Deep genetic structure and ecological divergence in a widespread human commensal toad. *Biology Letters*. **12**, 20150807 (2016). |
| Stöck et al. (2006) | M. Stöck, C. Moritz, M. Hickerson, D. Frynta, T. Dujsebayeva, V. Eremchenko, J. R. Macey, T. J. Papenfuss, D. B. Wake, Evolution of mitochondrial relationships and biogeography of Palearctic green toads (Bufo viridis subgroup) with insights in their genomic plasticity. *Molecular Phylogenetics and Evolution*. **41**, 663–689 (2006). |
| Zhang et al. (2016) | W. Zhang, X. Zhang, R. Guo, Y. Tang, Y. Zhang, The complete mitochondrial genome of *Bufo raddei*. *Mitochondrial DNA*. **27**, 3659–3660 (2016). |
| Stöck et al. (2008) | M. Stöck, A. Sicilia, N. M. Belfiore, D. Buckley, S. Lo Brutto, M. Lo Valvo, M. Arculeo, Post-Messinian evolutionary relationships across the Sicilian channel: Mitochondrial and nuclear markers link a new green toad from Sicily to African relatives. *BMC Evolutionary Biology*. **8**, 56 (2008). |
| Garcia-Porta et al. (2012) | J. Garcia-Porta, S. N. Litvinchuk, P. A. Crochet, A. Romano, P. H. Geniez, M. Lo-Valvo, P. Lymberakis, S. Carranza, Molecular phylogenetics and historical biogeography of the west-palearctic common toads (*Bufo bufo* species complex). *Molecular Phylogenetics and Evolution*. **63**, 113–130 (2012). |
| Özdemir et al. (2020) | N. Özdemir, C. Dursun, N. Üzüm, B. Kutrup, S. Gül, Taxonomic assessment and distribution of common toads (*Bufo bufo* and *B. verrucosissimus*) in Turkey based on morphological and molecular data. *Amphibia Reptilia*. **41**, 399–411 (2020). |
| Portik and Papenfuss (2015) | D. M. Portik, T. J. Papenfuss, Historical biogeography resolves the origins of endemic Arabian toad lineages (Anura: Bufonidae): Evidence for ancient vicariance and dispersal events with the Horn of Africa and South Asia. *BMC Evolutionary Biology*. **15**, 152 (2015). |
| Fontenot et al. (2014) | B. E. Fontenot, R. Makowsky, P. T. Chippindale, Nuclear-mitochondrial discordance and gene flow in a recent radiation of toads. *Molecular Phylogenetics and Evolution*. **59**, 66–80 (2011). |
| Brandvain et al. (2014) | Y. Brandvain, G. B. Pauly, M. R. May, M. Turelli, Explaining Darwin’s corollary to Haldane’s rule: The role of mitonuclear interactions in asymmetric postzygotic isolation among toads. *Genetics*. **197**, 743–747 (2014). |
| Thomé et al. (2010) | M. T. C. Thomé, K. R. Zamudio, J. G. R. Giovanelli, C. F. B. Haddad, F. A. Baldissera, J. Alexandrino, Phylogeography of endemic toads and post-Pliocene persistence of the Brazilian Atlantic Forest. *Molecular Phylogenetics and Evolution*. **55**, 1018–1031 (2010). |
| Fyhrquist et al. (1998) | N. Fyhrquist, K. Donner, P. A. Hargrave, J. H. McDowell, M. P. Popp, W. C. Smith, Rhodopsins from three frog and toad species: Sequences and functional comparisons. *Experimental Eye Research*. **66**, 295–305 (1998). |
| Pereyra et al. (2016) | M. O. Pereyra, D. Baldo, B. L. Blotto, P. P. Iglesias, M. T. C. Thomé, C. F. B. Haddad, C. Barrio-Amorós, R. Ibáñez, J. Faivovich, Phylogenetic relationships of toads of the *Rhinella granulosa* group (Anura: Bufonidae): A molecular perspective with comments on hybridization and introgression. *Cladistics*. **32**, 36–53 (2016). |
| Pauly et al. (2004) | G. B. Pauly, D. M. Hillis, D. C. Cannatella, The history of a nearctic colonization: Molecular phylogenetics and biogeography of the nearctic toads (*Bufo*). *Evolution*. **58**, 2517–2535 (2004). |
| Mendelson et al. (2011) | J. R. Mendelson, D. G. Mulcahy, T. S. Williams, J. W. Sites, A phylogeny and evolutionary natural history of mesoamerican toads (Anura: Bufonidae: *Incilius*) based on morphology, life history, and molecular data. *Zootaxa*. **3138**, 1–34 (2011). |
| Santos and Cannatella (2008) | J. C. Santos, D. C. Cannatella, Phenotypic integration emerges from aposematism and scale in poison frogs. *Proceedings of the National Academy of Sciences of the United States of America*. **108**, 6175–6180 (2011). |
| Vogel and Johnson (2008) | L. S. Vogel, S. G. Johnson, Estimation of Hybridization and Introgression Frequency in Toads (Genus: *Bufo*) Using DNA Sequence Variation at Mitochondrial and Nuclear Loci. *Journal of Herpetology*. **42**, 61–75 (2008). |
| Van Bocxlaer et al. (2009) | I. Van Bocxlaer, S. D. Biju, S. P. Loader, F. Bossuyt, Toad radiation reveals into-India dispersal as a source of endemism in the Western Ghats-Sri Lanka biodiversity hotspot. *BMC Evolutionary Biology*. **9**, 131 (2009). |
