# Supplementary material for: From Gondwana to the Yellow Sea, evolutionary diversifications of true toads Bufo sp. in the Eastern Palearctic and a revisit of species boundaries for Asian lineages
Source: eLife. 2022 Jan 28;11:e70494. doi: 10.7554/eLife.70494 (PMC8920510; doi:10.7554/eLife.70494)
Supplement: Transparent reporting form [file elife-70494-transrepform1.docx]

***eLife’s* transparent reporting form**

We encourage authors to provide detailed information *within their submission* to facilitate the interpretation and replication of experiments. Authors can upload supporting documentation to indicate the use of appropriate reporting guidelines for health-related research (see [EQUATOR Network](http://www.equator-network.org/%20)), life science research (see the [BioSharing Information Resource](https://biosharing.org/)), or the [ARRIVE guidelines](http://www.plosbiology.org/article/info:doi/10.1371/journal.pbio.1000412) for reporting work involving animal research. Where applicable, authors should refer to any relevant reporting standards documents in this form.

If you have any questions, please consult our Journal Policies and/or contact us: [editorial@elifesciences.org](mailto:editorial@elifesciences.org).

**Sample-size estimation**

- You should state whether an appropriate sample size was computed when the study was being designed
- You should state the statistical method of sample size computation and any required assumptions
- If no explicit power analysis was used, you should describe how you decided what sample (replicate) size (number) to use

Please outline where this information can be found within the submission (e.g., sections or figure legends), or explain why this information doesn’t apply to your submission:

All samples size for sequences data used for phylogeny, molecular dating, population dynamics and ecological niche modelling are adequate for robust analyses. The optimal sample size for tree topologies testing and species delimitation modelling are ensured by adequate power to detect statistical significance using marginal likelihood estimates (MLE), Bayes Factor and nested samplings.

The information on sample size is reported in several sections, matching with the analyses conducted in that section:

Results:

Phylogeny and population structure of East Asian *Bufo*

Materials and Methods:

Taxa sampling and dataset design

Species tree topology estimation

Phylogenetics and divergence dating of Asian Bufo

Past population dynamics

Estimation of migration patterns

Species delimitation modelling

Appendix 1

Supplementary file 1 –Supplementary files 1A-1O

**Replicates**

- You should report how often each experiment was performed
- You should include a definition of biological versus technical replication
- The data obtained should be provided and sufficient information should be provided to indicate the number of independent biological and/or technical replicates
- If you encountered any outliers, you should describe how these were handled
- Criteria for exclusion/inclusion of data should be clearly stated
- High-throughput sequence data should be uploaded before submission, with a private link for reviewers provided (these are available from both GEO and ArrayExpress)

Replicates/bootstraps and Markov chain Monte Carlo (MCMC) samplings are performed in the reconstruction of biogeography of Bufonidae using Maximum Likelihood and Bayesian Inference methods.

The adequacy for number of replicates/bootstraps in phylogeny and population genetic statistical analysis is determined by the resolution state of tree topology (fully resolved or not) and comparison of mean from calculated samples (between bootstraps 100-10000). Whereas, sampling efficacy for MCMC samplings in Bayesian-based analyses is diagnosed by the sampling convergence state, in which Effective Sample Size (ESS) number for each sampling parameter is ensured to reach 400 and above. The details information can be found in the following sections and subtopics:

Materials and methods:

Taxa sampling and dataset design

Section 1: Reconstruction of Holarctic bufonids biogeography

Section 2: Inferring patterns of diversification of Eastern Palearctic Bufo

Species tree topology estimation for Palearctic *Bufo*

Phylogenetics and divergence dating of Asian *Bufo*

Past population dynamics

Ecological niche modelling

Estimation of migration patterns

Species delimitation modelling

Supplementary files:

Caption Figure 3-figure supplement 1

Please outline where this information can be found within the submission (e.g., sections or figure legends), or explain why this information doesn’t apply to your submission:

**Statistical reporting**

- Statistical analysis methods should be described and justified
- Raw data should be presented in figures whenever informative to do so (typically when N per group is less than 10)
- For each experiment, you should identify the statistical tests used, exact values of N, definitions of center, methods of multiple test correction, and dispersion and precision measures (e.g., mean, median, SD, SEM, confidence intervals; and, for the major substantive results, a measure of effect size (e.g., Pearson's r, Cohen's d)
- Report exact p-values wherever possible alongside the summary statistics and 95% confidence intervals. These should be reported for all key questions and not only when the p-value is less than 0.05.

Please outline where this information can be found within the submission (e.g., sections or figure legends), or explain why this information doesn’t apply to your submission:

*N* taxa/populations per runs are described in these sections and subtopics:

Results:

Phylogeny and population structure of East Asian Bufo

Materials and methods:

Taxa sampling and dataset design

Phylogenetics and divergence dating of Asian *Bufo*

Past population dynamics

Estimation of migration patterns

Figures and figure legends:

Caption of Figure 2

Caption of Figure 3

Caption of Figure 4

Caption of Figure 4-figure supplement 1

Caption of Figure 5

Figure 6

(For large datasets, or papers with a very large number of statistical tests, you may upload a single table file with tests, Ns, etc., with reference to sections in the manuscript.)

**Group allocation**

- Indicate how samples were allocated into experimental groups (in the case of clinical studies, please specify allocation to treatment method); if randomization was used, please also state if restricted randomization was applied
- Indicate if masking was used during group allocation, data collection and/or data analysis

Please outline where this information can be found within the submission (e.g., sections or figure legends), or explain why this information doesn’t apply to your submission:

Sequence samples in phylogeny, demographic and population genetic analyses are allocated to specific groups based on geographic ranges and clades. This allocation is performed in AMOVA, STRUCTURE, Mantel Test, population dynamics and population migration. The grouping of sequences is described in

Results section, under subtopic of “Phylogeny and population structure of East Asian Bufo” and Materials and methods section, under subtopic of “Phylogenetics and divergence dating of Asian *Bufo”.*

The genetic samples and distribution data for Ecological Niche Modelling is allocated into two groups based on clades and geographic range. Group 1, southwestern Mainland (*Bufo gargarizans*) and Group 2, septentrional Eastern Asia (*B. sachalinensis*). The information can be found in Ecological Niche Modelling topic under Materials and Methods section.

**Additional data files (“source data”)**

- We encourage you to upload relevant additional data files, such as numerical data that are represented as a graph in a figure, or as a summary table
- Where provided, these should be in the most useful format, and they can be uploaded as “Source data” files linked to a main figure or table
- Include model definition files including the full list of parameters used
- Include code used for data analysis (e.g., R, MatLab)
- Avoid stating that data files are “available upon request”

Please indicate the figures or tables for which source data files have been provided:

The sequence data used in all analyses related to phylogeny and molecular dating of Holarctic and Eastern Palearctic Bufonidae are described in supplementary file 1J. All Genbank accession numbers of the sequences data can be referred in the same table.

The input files in the form of Beast code (.xml) generated from molecular dating analyses are deposited in Mendeley Data repository http://dx.doi.org/10.17632/wdtw6kn2t4.1 (Othman et al., 2021).

Additional data:

Molecular dating calibration discussed point by point in Appendix 1.

All supporting statistical values and data are provided as Supplementary file 1 - Supplementary files 1A-1O

The sources of references for huge datasets are further provided in Supplementary file 2
